# MoRNiNG: A Database of RNA Modification Sites Associated with RNA Secondary Structure Dynamics

**DOI:** 10.1093/gpbjnl/qzaf106

**Published:** 2025-11-14

**Authors:** Yicen Zhou (周奕岑), Shanxin Lyu (吕善鑫), Shiau Wei Liew (刘晓薇), Xi Mou (牟希), Ian Hoffecker, Jian Yan (严健), Yu Li (李煜), Chun Kit Kwok (郭骏杰), Jilin Zhang (张继林)

**Affiliations:** Department of Biomedical Sciences, College of Biomedicine, City University of Hong Kong, Hong Kong Special Administrative Region 999077, China; Department of Biomedical Sciences, College of Biomedicine, City University of Hong Kong, Hong Kong Special Administrative Region 999077, China; Department of Chemistry and State Key Laboratory of Marine Environmental Health, City University of Hong Kong, Hong Kong Special Administrative Region 999077, China; Department of Chemistry and State Key Laboratory of Marine Environmental Health, City University of Hong Kong, Hong Kong Special Administrative Region 999077, China; Department of Gene Technology, KTH Royal Institute of Technology, SciLifeLab, Solna 17165, Sweden; Department of Biomedical Sciences, College of Biomedicine, City University of Hong Kong, Hong Kong Special Administrative Region 999077, China; Tung Biomedical Sciences Centre, City University of Hong Kong, Hong Kong Special Administrative Region 999077, China; Department of Precision Diagnostic and Therapeutic Technology, City University of Hong Kong, Shenzhen Futian Research Institute, Shenzhen 518000, China; Department of Computer Science and Engineering, The Chinese University of Hong Kong, Hong Kong Special Administrative Region 999077, China; Department of Chemistry and State Key Laboratory of Marine Environmental Health, City University of Hong Kong, Hong Kong Special Administrative Region 999077, China; Shenzhen Research Institute of City University of Hong Kong, Shenzhen 518000, China; Department of Biomedical Sciences, College of Biomedicine, City University of Hong Kong, Hong Kong Special Administrative Region 999077, China; Tung Biomedical Sciences Centre, City University of Hong Kong, Hong Kong Special Administrative Region 999077, China; Department of Precision Diagnostic and Therapeutic Technology, City University of Hong Kong, Shenzhen Futian Research Institute, Shenzhen 518000, China

**Keywords:** RNA G-quadruplex, RNA modification, RNA secondary structure, RNA-binding protein, Conformation dynamics

## Abstract

RNA structures are essential building blocks of functional RNA molecules. Profiling secondary structures *in vivo* and in real time remains challenging because RNAs exhibit dynamic structures and complex conformations. In addition to the canonical stem-loop secondary structure, the non-canonical RNA G-quadruplex (rG4) structure has attracted interest for its potential as a drug target. Early studies have demonstrated that RNAs can form distinct secondary structures. However, how distinct RNA structures formed from the same RNA sequence function within the transcriptome is poorly understood, and the factors that drive and regulate structural transitions remain to be investigated. Inspired by the ability of a *HOXB9* segment to form multiple structures, we found that many RNA segments across the transcriptome exhibit multi-faceted structure-forming potential. In the case of *HOXB9*, we demonstrated that *N*^6^-methyladenosine (m^6^A) modification influences RNA structure and binding to RNA-binding proteins (RBPs). Therefore, we collected RNA modification sites naturally occurring within the putative G-quadruplex-forming sequences (PQSs) of transcripts and developed MoRNiNG, a database for RNA modifications in natural rG4 structures. MoRNiNG is organized into reliability tiers determined by the resolution of RNA modification sites and is designed to accommodate various large datasets. We experimentally validated the influence of m^6^A, 5-methylcytosine (m^5^C), and adenosine-to-inosine (A-to-I) editing on rG4-forming sequences, providing evidence to support the modification switch concept. The diversity and transition of secondary structures from the same RNA segment offer valuable insights into the regulation of RNA structural dynamics. MoRNiNG is freely accessible at https://www.cityu.edu.hk/bms/morning.

## Graphical abstract

**Figure:**
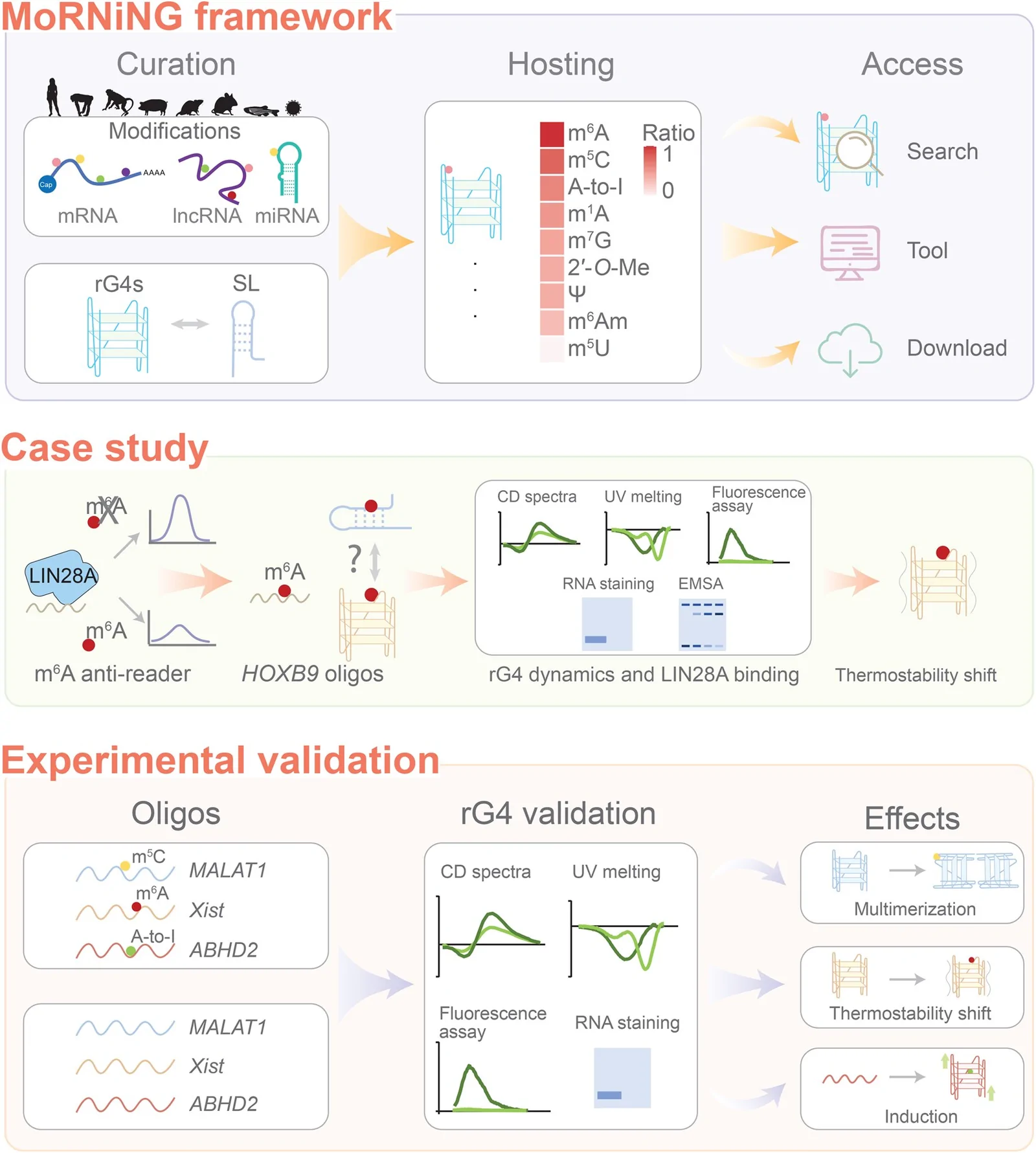

## Introduction

RNA molecules are instrumental in many biological processes [1]. Understanding their functional roles is crucial for constructing a comprehensive regulatory map. One of the significant challenges hindering this process is the elusive information that determines the transition between linear sequences and structural RNAs, as well as among different RNA structures.

RNAs form a variety of structures, and a growing volume of RNA profiling data has demonstrated that many bases participate in canonical base pairing [2,3]. Beyond canonical secondary RNA conformations, such as stem-loop (SL) structures, internal loops, and multi-branch junctions, the non-canonical structure RNA G-quadruplex (rG4) can form when RNA strands fold to stack more than two planar G-tetrads, with each tetrad consisting of four guanines (Gs) connected via Hoogsteen hydrogen bonds and stabilized by a monovalent cation [4,5]. Since its discovery, the rG4 structure has gained recognition as a potential functional element in both transcriptional and post-transcriptional regulation. Numerous assays have been devised and refined to profile rG4s *in vitro* and *in vivo* [4,5], offering unprecedented transcriptome-wide insights into the distribution and function of rG4s. However, the rG4 landscape and dynamics require substantial exploration to gain a comprehensive understanding.

The flexible nature of single-stranded RNAs allows them to dynamically change conformation to interact with RNA-binding proteins (RBPs) with structural specificity [6,7]. A typical *in vivo* example is LIN28A, which binds to the pre-*let-7* terminal SL structure through its cold shock domain to inhibit the function of miRNA *let-7* [8]. This protein can also unwind rG4 in mRNAs to promote ribosomal scanning and enhance translation [9]. The polycomb repressive complex 2 (PRC2) interacts with rG4 in *Xist*, which is crucial to silence genes on chromosome X in a stepwise manner [10]. These cases emphasize the importance of structural RNA in regulating gene expression. In addition to previously reported synthetic ligands, evidence suggests that a single RNA sequence can adopt multiple distinct structures, linking the biological function of RNA *in vivo* to structural specificity [11,12]. Moreover, the *N*^6^-methyladenosine (m^6^A) modification at the LIN28A-binding site on *HOXB9* mRNA can reduce the binding affinity of LIN28A, suggesting a potential alteration in RNA secondary structure. As RNA structures across cellular states remain incompletely characterized and the factors driving conformational changes are still poorly understood, further investigation is needed to elucidate transitions between RNA secondary structures.

RNA modifications, such as m^6^A and 5-methylcytosine (m^5^C), are chemical changes that can alter the properties of local RNA sequences and their corresponding RNA structures [13–16]. Indeed, these modifications play vital roles in regulating the formation and stability of rG4s [17,18]. In the case of miRNA *let-7*, *N*^7^-methylguanine (m^7^G) modification changes the equilibrium between SL and rG4, enabling DROSHA binding and promoting miRNA maturation [19]. Notably, the key component of the methyltransferase complex, METTL14, prefers binding to rG4s [18], underscoring the role of rG4s in regulating m^6^A-dependent biological processes. Yet, the impact of RNA modifications on RNA secondary structures remains poorly characterized and requires further investigation. Despite the need to increase the spectrum and resolution of RNA modification profiling, gaining a large volume of rG4 profiles still requires substantial effort. The emerging consequences of RNA modifications in reshaping RNA structures should also be considered to gain deeper insights into RNA function.

To bridge the knowledge gap between RNA epigenetics and structural dynamics, we present Modification of RNAs in Natural rG4 (MoRNiNG), a database with a curated compendium of rG4-forming sequences containing various cellular RNA modification sites, partially supported by experimental evidence. Although the diversity of rG4s is not clearly defined at this stage, we provide evidence supporting the observation that RNA modifications can modulate rG4 structures. The database design is structured to accommodate future expansion and ensure compatibility with diverse data types, laying the foundation for a more comprehensive understanding of RNA structural dynamics.

## Data collection and processing

### Annotation of rG4 and PQS

Three types of rG4 datasets were included to characterize transcriptome-wide rG4 features or identify potential rG4 loci. These datasets include *in vivo* experimental data such as rG4-seq and ultra-low-input rG4-seq [20–23], *in vitro* circular dichroism (CD) spectra [24], and rG4 prediction results generated by several computational tools, including pqsfinder [25], QGRS Mapper [26], G4Hunter (G4H) [27], G4RNA screener [28], RNAfold [29], G4 Neural Network (G4NN; score > 0.5), and the Quadparser-based G_2_L_12_ motif [defined as (G_≥2_N_1–12_)_3_G_≥2_] [30]. As the detection capacity and scope of *in vivo* approaches are usually limited, we applied a hierarchical prediction scheme to obtain consensus rG4 loci, thereby strengthening reliability and reducing false-positive loci. Following the primary predictions using pqsfinder [25] and QGRS Mapper [26], the extracted consensus putative G-quadruplex-forming sequences (PQSs) were further examined with additional prediction tools and validated by experimental evidence to enhance reliability. This process resulted in annotated tags based on the evidence sources (File S1). Additionally, putative GI-quadruplex-forming sequences (PIQSs), a type of G-quartet induced by the substitution of G with inosine (I) through RNA editing in the human transcriptome, were included [31]. As not all PIQSs predicted by pqsfinder (score ≥ 10) originated from the longest transcripts, a separate “modType” option was introduced on the search page.

The distribution of all PQSs within mRNAs and long non-coding RNAs (lncRNAs) was visualized using the Guitar R package [32], with the annotation of 5′ untranslated region (UTR), coding sequence (CDS), and 3′ UTR. The number of PQSs per transcript was normalized using the following formula: normalized count = (PQS count × 1000) / transcript length. Kernel density estimation was performed using a Gaussian kernel.

### Prediction of SL structure embedded in PQSs

PQSs in the longest transcripts, including mRNAs and lncRNAs annotated in the Ensembl database, miRNAs shared between the Ensembl and miRBase databases [33], and all remaining RNA types (Others), were subjected to RNAfold [29] to predict the canonical SL structures.

### Collection of RNA modification sites

To obtain a panoramic view of the RNA modification dataset associated with genes, nine types of modification sites were collected, including m^6^A, m^5^C, 5-methyluridine (m^5^U), *N*^6^,2′-*O*-dimethyladenosine (m^6^Am), m^7^G, *N*^1^-methyladenosine (m^1^A), 2′-*O*-methylation (2′-*O*-Me), adenosine-to-inosine (A-to-I) editing, and pseudouridine located within exons of the longest transcripts. Data were sourced from the Gene Expression Omnibus (GEO), RMBase, RMVar, MiREDiBase, m^6^A-Atlas, and other public datasets [34–37], covering nine species to construct a database illustrating the interplay between RNA modifications and rG4. Modification sites annotated on older genome assemblies were converted to the latest University of California, Santa Cruz (UCSC) or National Center for Biotechnology Information (NCBI) genome versions using LiftOver (**Table 1**), including human (UCSC hg38), mouse (UCSC mm39), rat (UCSC rn7), rhesus monkey (UCSC rheMac10), chimpanzee (UCSC panTro5), pig (UCSC susScr11), zebrafish (UCSC danRer11), human immunodeficiency virus type 1 (HIV-1; NCBI NC_001802.1), and simian virus 40 (SV40; NCBI NC_001669.1). Gene annotations of corresponding genomes were downloaded from the Ensembl database (release 109). Transcript annotations of mRNA and lncRNA from Ensembl and miRNA from miRBase (v22) were considered [33]. The modification sites of the two RNA viruses were based on their genomic RNA or mRNA.

**Table 1:** Data sources and summary of studied species

Species	Genome	Annotation	No. of modifications on transcripts	No. of unique transcripts	No. of unique PQSs on transcripts
Human	UCSC hg38	Ensembl (release 109)	m^6^A: 34,527 m^1^A: 515 m^5^C: 5596 m^7^G: 208 m^5^U: 3 m^6^Am: 59 2′-*O*-Me: 163 Ψ: 103 A-to-I editing: 1165	mRNA: 12,400 lncRNA: 3057 pre-miRNA: 18 miRNA: 11	35,189
Mouse	UCSC mm39	Ensembl (release 109)	m^6^A: 25,153 m^1^A: 3 m^5^C: 2041 m^7^G: 19 Ψ: 38 A-to-I editing: 123	mRNA: 10,304 lncRNA: 1102 pre-miRNA: 4 miRNA: 1	23,666
Rhesus monkey	UCSC rheMac10	Ensembl (release 109)	m^6^A: 2225	mRNA: 1474	1994
Chimpanzee	UCSC panTro5	Ensembl (release 109)	m^6^A: 2521	mRNA: 1665 lincRNA: 2	2254
Pig	UCSC susScr11	Ensembl (release 109)	m^6^A: 7899	mRNA: 4334 lncRNA: 11	7130
Rat	UCSC rn7	Ensembl (release 109)	m^6^A: 4320 m^1^A: 1 m^5^C: 1 m^7^G: 1 Ψ: 2	mRNA: 2690 lncRNA: 12	3874
Zebrafish	UCSC danRer11	Ensembl (release 109)	m^6^A: 1373	mRNA: 1032 lincRNA: 6	1249
HIV-1	NCBI NC_001802.1		m^6^A: 2	Genomic RNA: 2	2
SV40	NCBI NC_001669.1		m^6^A: 1	Late mRNA: 1	1

*Note*: UCSC, University of California, Santa Cruz; NCBI, National Center for Biotechnology Information; PQS, putative G-quadruplex-forming sequence; m^6^A, *N*^6^-methyladenosine; m^1^A, *N*^1^-methyladenosine; m^5^C, 5-methylcytidine; m^7^G, *N*^7^-methylguanosine; m^5^U, 5-methyluridine; m^6^Am, *N*^6^,2′-*O*-dimethyladenosine; 2′-*O*-Me, 2′-*O*-methylation; Ψ, pseudouridine; A-to-I editing, adenosine-to-inosine RNA editing; HIV-1, human immunodeficiency virus type 1; SV40, simian virus 40; mRNA, messenger RNA; miRNA, microRNA; lncRNA, long non-coding RNA; lincRNA, long intergenic non-coding RNA.

RNA modification sites were categorized into three confidence levels: high, medium, and low, according to experimental or prediction methods (Table S1). The high confidence level reflects modification sites detected at single-nucleotide resolution with experimental evidence, while the low confidence level denotes RNA modifications derived from either databases or predictions. Information regarding the source of modification sites and corresponding publications is incorporated into detailed pages in the MoRNiNG database (Table S2).

### Data processing and analysis in the database

RNA modification sites and candidate rG4 loci were processed to obtain base-resolution RNA modification sites located within rG4 or potential rG4-forming sequences using BEDTools [38]. Each modification site was assigned a unique ID following the nomenclature format: species_modification_unique identifier. The locations of modification sites were further classified into loop, G-tetrad, and bulge to reflect their positions within the structures.

## Database implementation and content

### Database implementation

The database was implemented using SQL Server to store RNA modifications and PQSs from nine species in separate tables at the back end. The web interface was developed using Hypertext Markup Language (HTML), Cascading Style Sheets (CSS), and JavaScript (JS). Active Server Pages (ASP) scripts were employed to query the SQL Server back-end database through JS by AJAX forms or Fetch API. For real-time statistical information, interactive plots were created by ECharts or D3.js. To display the detailed information of each genomic locus, an embedded JBrowse [39] was inserted into the corresponding subpages with a focused view at the selected locus.

### Database content

**Figure 1:**
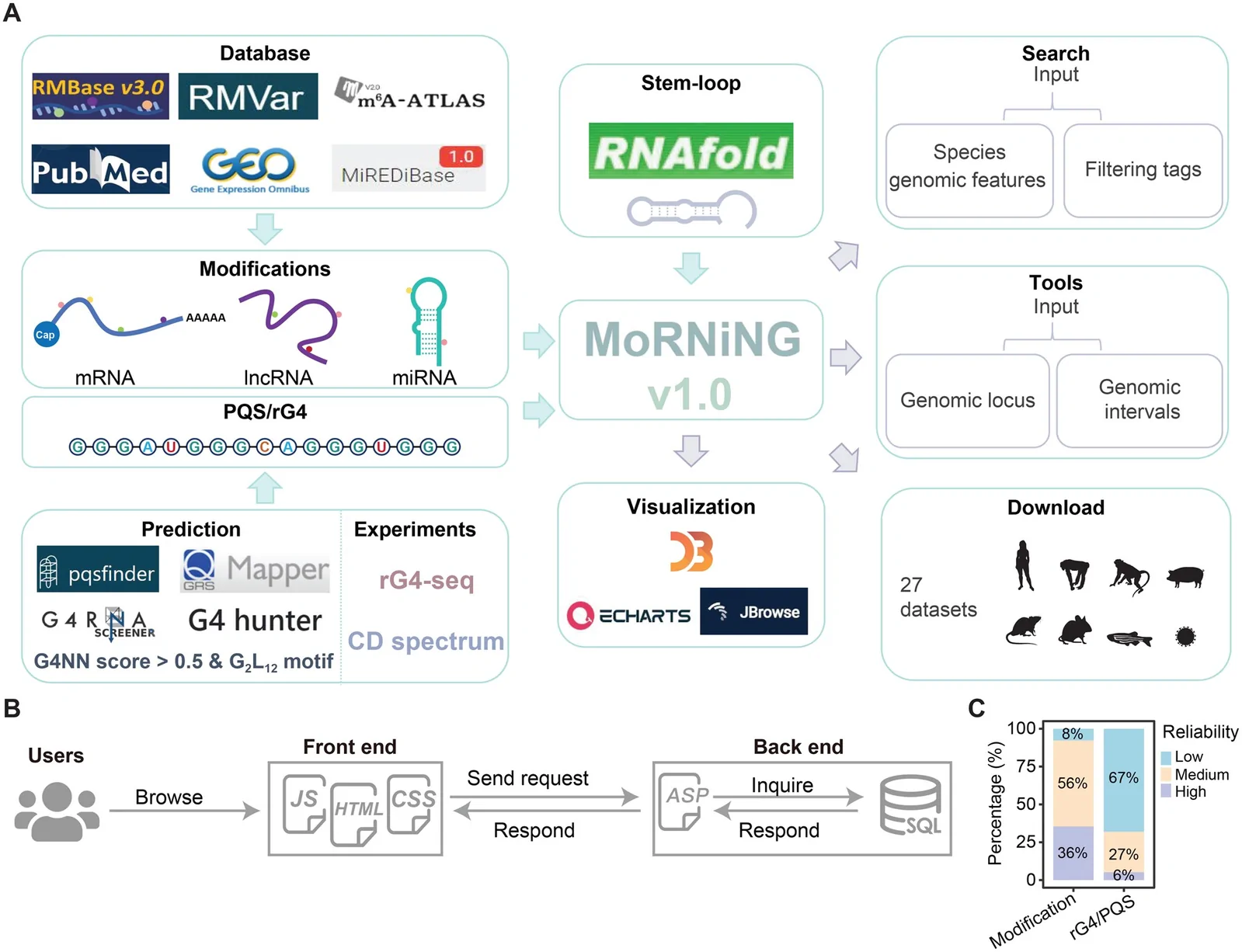
Architecture of MoRNiNG **A**. Schematic illustration of the data sources, approaches, and functions of the MoRNiNG database. The sources of RNA modifications, tools, and experiments for rG4s and PQSs are shown in the left panel, while the major functions are presented in the center and right panels. The tools and datasets are summarized on the right. **B**. Architecture and modular implementation of the front and back ends. The major techniques used to implement front-end and back-end functions are depicted in groups. **C**. Reliability level of RNA modification sites and rG4s/PQSs cataloged in the database. MoRNiNG, Modification of RNAs in Natural rG4; lncRNA, long non-coding RNA; miRNA, microRNA; rG4, RNA G-quadruplex; PQS, putative G-quadruplex-forming sequence; CD, circular dichroism; JS, JavaScript; HTML, Hypertext Markup Language; CSS, Cascading Style Sheets; ASP, Active Server Pages; SQL, Structured Query Language.

In the database, we reported RNA modification loci within rG4s validated by experiments or PQSs predicted by several rG4 prediction tools. We collected nine types of RNA modifications from nine species to demonstrate the preference for modified sites in the transcriptome (**Figure 1**A and B; Table 1). The primary function of this database is to host RNA modification loci with tiered reliability, enabling researchers to explore the relationship between RNA modifications and rG4s and to reveal their potential biological impacts. To our knowledge, this dataset is a dedicated collection linking canonical and non-canonical RNA structures in natural RNAs. This work also proves that RNA modifications can alter rG4-forming propensity, which may play a role in post-transcriptional regulation.

**Figure 2:**
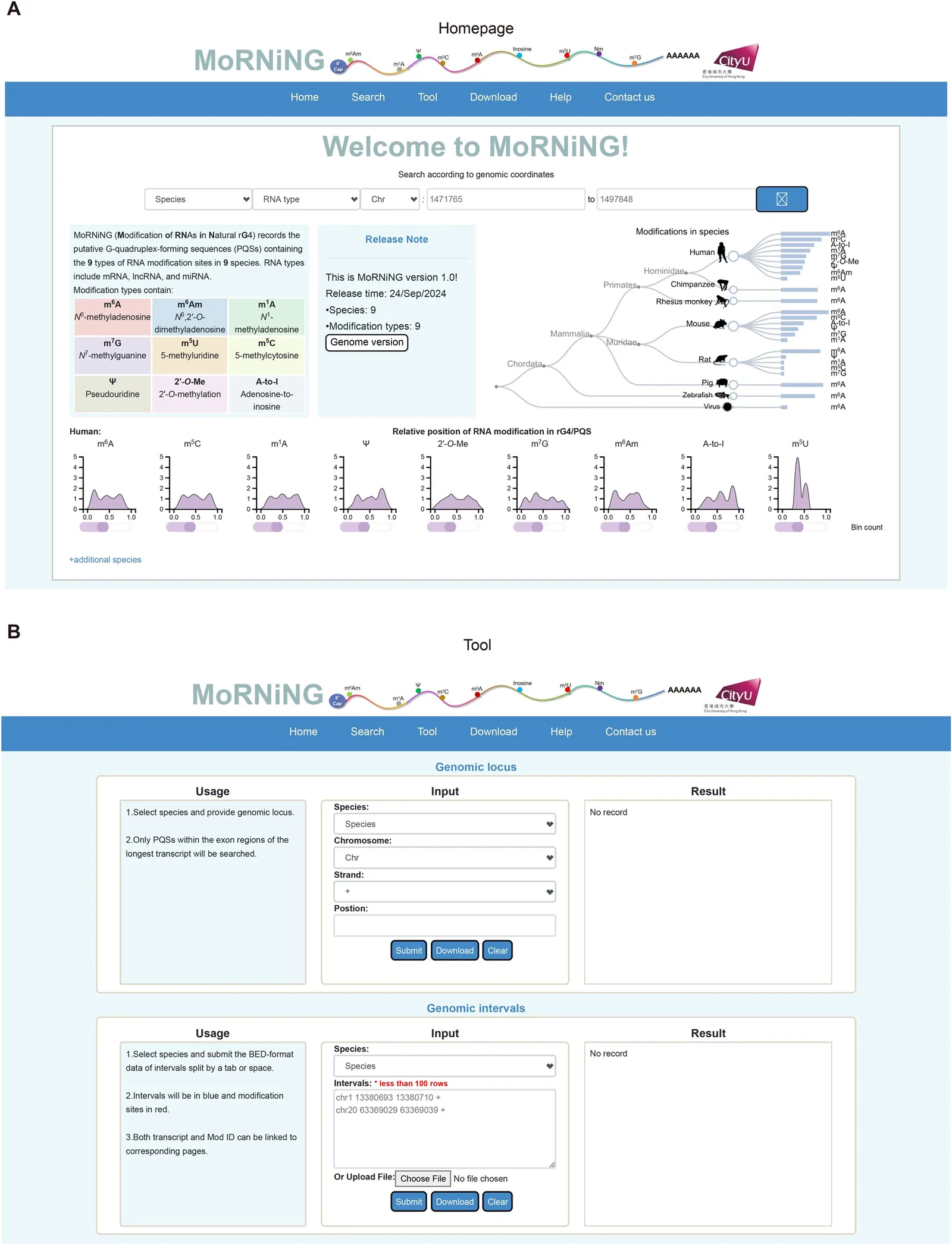
Interface and major functions of MoRNiNG **A**. Snapshot of the main page. **B**. Snapshot of the tool function.

The MoRNiNG homepage features a real-time tree plot visualizing the constituent species and the number of cataloged RNA modification sites (**Figure 2**A). The positional preference plot of pronounced modification sites in each cataloged species is visualized. We implemented three primary pages, including search, download, and tool (Figure 2B, Figure S1), for users to navigate to distinct functions. A help page is offered to provide usage instructions, updates, and information on the corresponding data sources.

### User interface

#### Browsing interface

The main page of the MoRNiNG database provides a summary of the total number of RNA modification sites in rG4s/PQSs across the studied species. A search engine is embedded within the interface, allowing users to access the database by specifying the species and genomic locus. The search page provides multiple filters under eight categories, including species, chromosome, modification type, RNA type, and four additional options. Users can combine different filtering tags within each species to retrieve desired RNA modifications associated with rG4s. The tool page offers two major functions that allow users to upload genomic loci or regions of interest with RNA modifications, either in locus-specific or batch mode. On the download page, formatted tables for each species can be downloaded.

#### Tiered reliability and interactive visualization

We classified RNA modification sites into three reliability tiers based on the resolution of corresponding experimental evidence or predictions. Loci in the high-reliability tier are supported by experiments at single-nucleotide resolution. Sites identified by methods that detect regional RNA modifications are classified as medium-reliability, while sites that are fully predicted or derived from the DARNED [40], RADAR [41], and MODOMICS [42] databases are assigned to the low-reliability tier. PIQSs predicted by pqsfinder with scores ≥ 10 are also assigned to the low-reliability tier. The reliability information of sites supported by multiple sources is fully presented. Similarly, we introduced a system with tiered reliability to rG4s/PQSs: loci with experimental evidence or supported by more than two prediction tools are labeled as high-reliability; loci supported by one additional prediction tool besides pqsfinder and QGRS Mapper are classified as medium-reliability; and loci in the low-reliability tier are those either supported by the consensus sequence predicted by pqsfinder and QGRS Mapper or obtained through the criteria of G4NN score > 0.5 and the G_2_L_12_ motif (Figure 1C). Each RNA modification site includes modification type, genomic position, strand, gene symbol, Ensemble transcript ID, rG4/PQS coordinate, relative modification position within rG4/PQS, site type, detection method, and data source (Figure S2). An interactive genome browser, JBrowse, is embedded to visualize the rG4s within the annotated transcripts.

#### Tools for user-provided genomic loci or intervals

MoRNiNG provides web tools for preliminary analysis, including the extraction of RNA modification sites from experimentally validated rG4-forming loci and the identification of RNA modification-associated PQSs. These two tools allow users to obtain rG4s/PQSs or RNA modification sites from the longest transcript of each gene (Figure S3). Each search job is executed instantly, and the results are visualized upon job completion.

#### Data download

All processed RNA modifications within rG4s of distinct RNA species can be downloaded through the browse page after applying selection criteria. Comprehensive modification sites and their information stored as zip files can be accessed on the download page for each species. The results generated by querying user-provided genomic loci can also be downloaded.

## Case study

### RNA modification affects the RNA secondary structure that interacts with LIN28A

**Figure 3:**
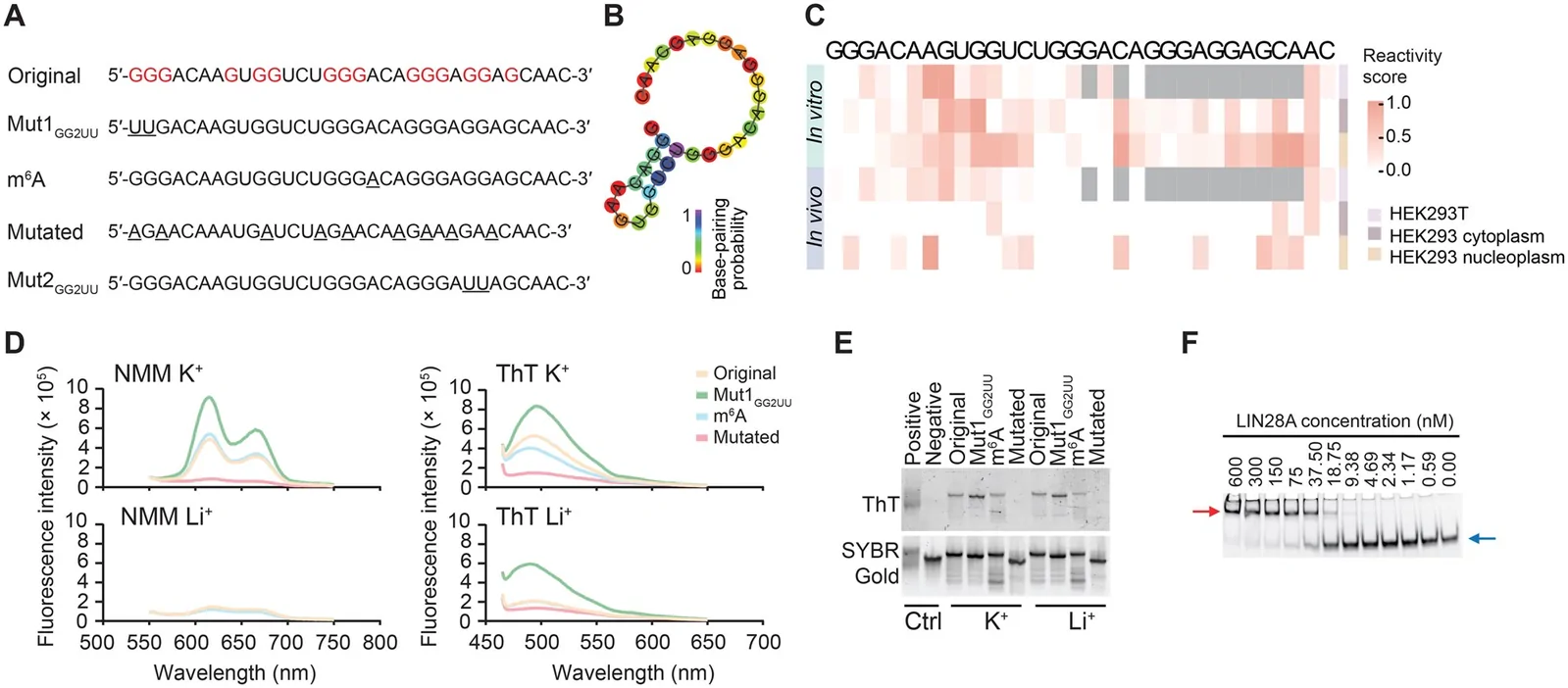
Structural diversity of the *HOXB9* mRNA segment **A**. Sequences of the synthesized original *HOXB9* RNA oligo and its derivatives with mutations and m^6^A modification. Potential G-tetrads are highlighted in red in the original oligo, while UU replacement, m^6^A modification, and mutations in the fully mutated sequence are displayed with underlines. **B**. Predicted canonical SL structure of the original *HOXB9* RNA oligo by RNAfold. The base-pairing probability is visualized in color. **C**. Diverse icSHAPE reactivity scores of the original *HOXB9* RNA oligo. *In  vivo* and *in vitro* profiles were captured from HEK293T cells and the cytoplasm and nucleoplasm of HEK293 cells. The reactivity score ranges from 0 to 1, with higher scores indicating greater single-strandedness. **D**. Fluorescence assay using G4-specific ligands ThT and NMM under K^+^ and Li^+^ conditions, respectively. **E**. rG4 formation of the original *HOXB9* RNA oligo and its derivatives stained by ThT and SYBR Gold. The conditions used to stabilize and destabilize rG4 are shown. The rG4-forming TERRA RNA and G-tetrad-mutated TERRA RNA were used as rG4 positive and negative controls, respectively. **F**. EMSA of the original *HOXB9* RNA oligo (10 nM, FAM-labeled) with increasing concentrations of LIN28A (0–600 nM) under K^+^ condition. Red and blue arrows indicate the LIN28A–RNA complex and free probe, respectively. SL, stem-loop; NMM, *N*-methylmesoporphyrin IX; ThT, Thioflavin T; TERRA, telomeric repeat-containing RNA; EMSA, electrophoretic mobility shift assay; FAM, fluorescein amidite; Ctrl, control.

As RNA modifications participate in regulating the dynamic interactions between RBPs and RNA structures, we investigated the binding sites of RBP LIN28A, which acts as an m^6^A anti-reader in mouse embryonic stem cells [17]. We discovered that one SL-forming RNA segment in *HOXB9*, documented in the MoRNiNG database and targeted by LIN28A, could also form an rG4, as predicted (**Figure 3**A and B). The prediction based on reactivity score, however, only indicated potential SLs **(**Figure S4A and B). Additionally, the profiles captured *in vivo* and *in vitro* did not align well within G-stretches, deviating from RNAfold prediction [29]. This shows that RNA structure prediction by RNAfold does not fully account for solution properties that could affect RNA folding, such as surrounding ions (Figure 3C). In the structure probing datasets, G-stretches are more frequently protected than other bases in PQSs, as Gs in G-tetrads show a statistically lower reactivity score than bases in the loop in several cell lines, including H9, HEK293, HeLa, HepG2, and K562 (Figure S4C). Although G tends to be more reactive in structure probing assays and the reactivity score reflects the structural average, G-tetrad scores are significantly lower than those of Gs in non-PQS regions (Figure S4D), implying that G-tetrads are generally protected in PQSs. To confirm the rG4 formation of this *HOXB9* RNA segment, synthesized RNA oligos were examined. A fully mutated sequence disrupting intramolecular rG4 formation was designed as a negative control. To demonstrate that rG4 can form from a sequence that also folds into an SL, we further designed two mutated sequences, Mut1_GG2UU_ and Mut2_GG2UU_, based on the original oligo with the replacement of two bases, GG to UU, at the 5′ end and 3′ end, respectively (Figure 3A). Indeed, fluorescence assays using G4-specific ligands *N*-methylmesoporphyrin IX (NMM) and Thioflavin T (ThT) confirmed rG4 formation in the presence of K^+^ (Figure 3D). In addition, the rG4 formed *in vitro* exhibited a complex composition according to the electrophoresis and fluorescence assays, as this RNA oligo contains G-tetrads capable of forming two types of rG4. The comparison of rG4s stained by G4-specific ligand ThT and total RNA stained with SYBR Gold provided strong evidence that both the original and Mut1_GG2UU_ sequences form rG4s (Figure 3E; Table S3). The original oligo likely forms several distinct rG4 structures in solution, given that several types of PQSs were predicted (Figure S4A), and the Mut1_GG2UU_ sequence yielded a much stronger signal in the fluorescence assay (Figure 3D). The CD and UV melting results for Mut2_GG2UU_ further confirmed the formation of compound rG4s (Figure S5). This finding is consistent with the observation that compound RNA conformations in solution exhibit lower rG4 absorption than pure rG4 conformation [43] (Figure S6).

Because m^6^A modification occurs naturally in this RNA segment, we used a synthesized oligo carrying an m^6^A modification to investigate rG4 formation. ThT staining demonstrated that m^6^A reduces the rG4-forming potential under Li^+^ conditions that destabilize rG4 (Figure 3E). This reduced rG4 stability is consistent with the results of UV melting experiment, which showed that m^6^A decreases rG4 thermostability (Figure S7). The respective rG4 melting temperatures (Tms) of RNAs with and without m^6^A were 55°C and 55.5°C in the forward scan, and 51°C and 53°C in the reverse scan. This suggests that RNA modifications affect rG4 folding.

Next, we examined the binding between rG4 and LIN28A to assess the influence of RNA modifications (Figure 3F, Figure S8). Despite their binding in the presence of K^+^, we discovered that the RNA carrying m^6^A showed no significant difference in binding affinity to LIN28A under the rG4 stabilizing condition compared to the RNA without m^6^A modification. This suggests that m^6^A potentially affects rG4 under conditions in which rG4 can easily unwind or compete with other conformations. The binding affinity between m^6^A-carrying RNA and LIN28A indeed declined under the Li^+^ condition (Figure S8). The comparison of electrophoretic mobility shift assay (EMSA) results under K^+^ and Li^+^ conditions indicates that rG4 enhances RNA binding to LIN28A. LIN28A could also bind to alternative RNA sequences or conformations, potentially influenced by K^+^. This finding aligns with the observation that m^6^A can slightly lower the rG4 Tm. This mild change enables the conformation to shift toward SL, as indicated by the ThT fluorescence assay (Figure 3D). The m^6^A-induced structural change can explain the decreased binding affinity between RNA and LIN28A when rG4 is the preferred binding conformation. Therefore, LIN28A can be captured as an m^6^A anti-reader in loci where rG4 and SL are nested. These results demonstrate that m^6^A can affect the conformation of rG4-containing sequences and their interactions with LIN28A.

### Genome-wide existence of RNAs with multiple-structure forming potential

**Figure 4:**
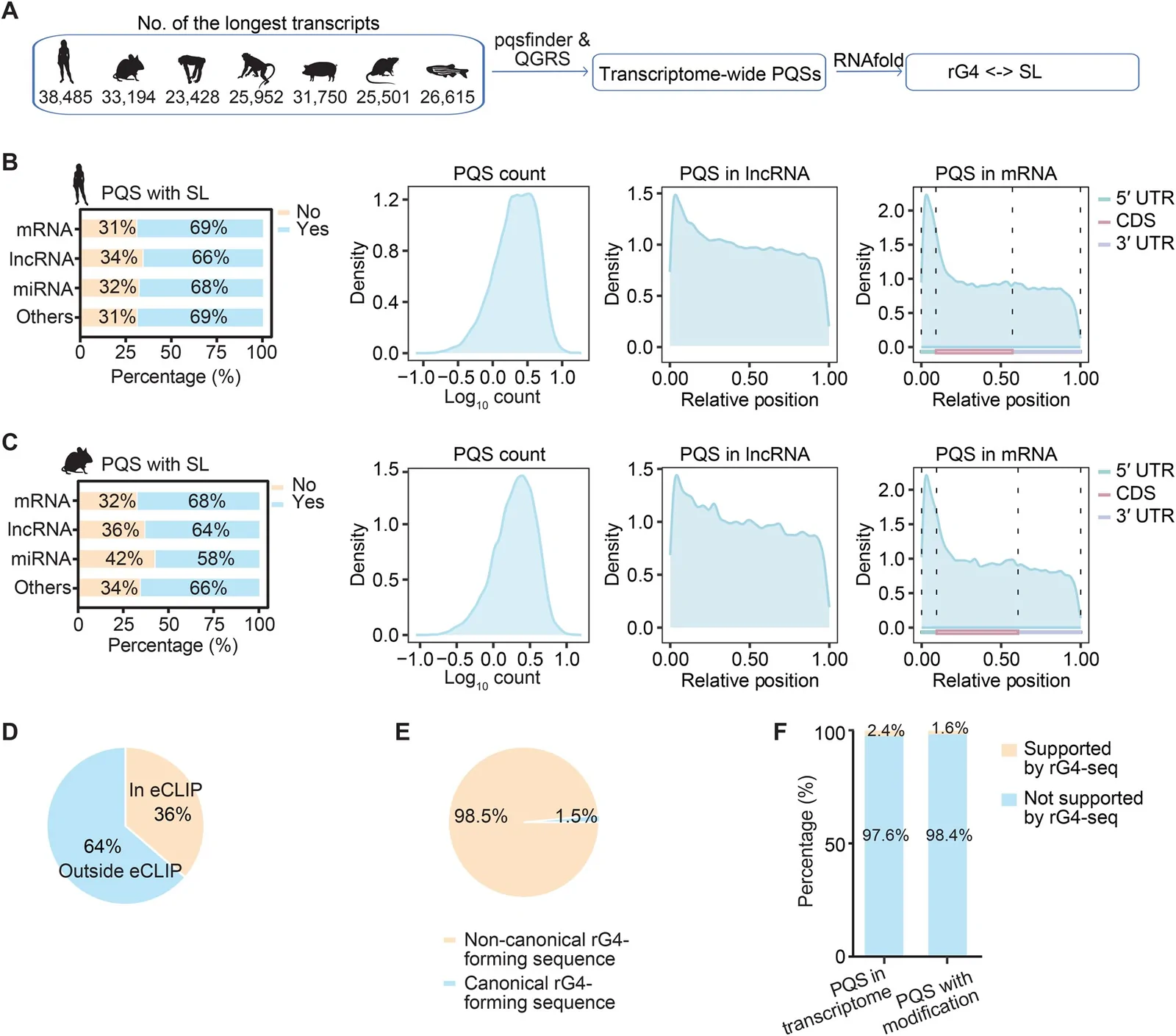
Dual structure-forming nature of RNA segments in transcripts **A**. Workflow to collect PQSs with SL-forming potential in the transcriptome of vertebrate genomes, including human, chimpanzee, rhesus monkey, pig, mouse, rat, and zebrafish. **B**. SL-forming potential and distribution of PQSs in the human transcriptome. Left: Percentage of PQSs predicted to form SLs in mRNAs, lncRNAs, miRNAs, and the remaining RNAs in the human transcriptome. Middle-left: The distribution of PQS count per human transcript, normalized for length to counts per kilobase. Middle-right: The relative position of human PQSs in the longest lncRNAs. Right: The relative position of human PQSs in the longest mRNAs. **C**. SL-forming potential and distribution of PQSs in the mouse transcriptome. **D**. Percentage of PQSs supported by ENCODE eCLIP peaks in human sequences. **E**. Percentage of canonical and non-canonical rG4-forming sequences lacking SL structure in the human transcriptome. **F**. *In vivo* evidence for PQSs and PQSs with RNA modifications. Left: Percentage of PQSs supported by rG4-seq in the human transcript. Right: Percentage of PQSs with RNA modifications supported by rG4-seq in the human transcript. UTR, untranslated region; CDS, coding sequence.

Rather than recording comprehensive maps of RNA structural dynamics, current RNA structure profiling assays, including SHAPE-Seq, icSHAPE, DMS-MaPseq, and rG4-seq [20,44–46], primarily provide static snapshots of structural landscapes in a few experimental settings. It remains largely unknown how distinct secondary structures formed from the same locus undergo transition. We combined computational predictions and public datasets to investigate the potential transitions between distinct RNA structures. We focused on SL and rG4 structures because of their prevalence and increasingly available experimental datasets. We extracted PQSs from the longest transcripts of mRNA, lncRNA, and miRNA based on consensus PQSs predicted by pqsfinder [25] and QGRS Mapper [26], followed by an SL structure analysis using RNAfold [29] in six mammalian and one fish species (**Figure 4**A). A relatively loose threshold was applied to include non-canonical PQSs (File S1). By screening the human and mouse transcriptomes, we observed that at least 60% and 50% of PQSs, respectively, can form SLs across distinct RNAs. This was predicted by RNAfold [29] based on the minimum free energy (MFE) ensemble (Figure 4B and C), implying a widespread existence of embedded secondary RNA structures. The MFE distribution of canonical PQSs was significantly different from that of imperfect PQSs (Figure S9), indicating a relatively flexible nature of imperfect PQSs. Although G-tetrad demands G-stretches and G can pair with uracil (U) in nature [47], the stem of SL is more likely to appear in the loops of PQSs. By analyzing the number and occurrence of PQSs on mRNA and lncRNA in the human transcriptome, the normalized density of PQS counts based on relative positions revealed an uneven distribution of PQSs at both transcript ends. The number of PQSs in each transcript varied significantly, ranging from 1 to 346, suggesting that transcripts use rG4 distinctly (Figure 4B). A similar pattern was observed in the transcriptomes of mice and other species, except for zebrafish (Figure 4C, Figure S10). These results imply the widespread existence of secondary structure equilibrium in transcriptomes.

### PQSs and the scarcity of experimental rG4 profiles in the human transcriptome

About 36% of PQSs in the human transcriptome are supported by at least one eCLIP dataset from ENCODE [48], consistently demonstrating that RBPs can interact with rG4s *in vivo* (Figure 4D). These PQS-overlapping peaks, which span at least half the length of the corresponding PQSs, were used to identify candidate rG4-binding RBPs. These rG4-binding RBPs were defined as proteins that bind PQS-overlapping peaks, which account for at least 15% of the total non-transposable element (TE) peaks in this study. The functional enrichment of rG4-binding RBPs showed significant overrepresentation in splicing, nucleoprotein granules, and stress granules (Figure S11). This result aligns with previous discoveries of co-localization between rG4s and ribonucleoproteins [49,50]. Notably, among the PQSs that RNAfold failed to predict SL structures, we noticed that canonical rG4s (G_3_, L_1-7_) and non-canonical rG4s accounted for 1.5% and 98.5%, respectively (Figure 4E). This distribution significantly differed from that of PQSs predicted to form SL. Furthermore, based on rG4-seq and rG4-seq (v2.0) data from the human transcriptome, we analyzed PQSs that either completely overlap with rG4-seq peaks or have their last G at reverse transcriptase stalling (RTS) sites or 1 nt upstream, and found that only about 2.4% of PQSs were supported by rG4-seq (Figure 4F). Additionally, the current rG4-seq dataset solely includes data from HeLa and HEK293T cells, highlighting the necessity to generate more rG4 profiles to investigate the dynamics of distinct RNA secondary structures. To demonstrate the reliability of PQSs, CD-HIT-EST [51] was employed to cluster PQSs collected in this study with the reference PQSs documented in QUADRatlas [52] and G4Atlas [53]. Over 69% of the reference PQSs were clustered with the collected PQSs at a 90% sequence identity threshold; this increased to 87% when the identity threshold was set to 85% (Figure S12A). We additionally applied two large language models, RNA-FM [54] and RNAErnie [55], to compare feature similarity between PQS datasets using two PIWI-interacting RNA (piRNA) datasets as outgroups. The two PQS datasets showed good alignment in the projected space after dimensionality reduction, indicating their high similarity (Figure S12B). In contrast, the piRNA sequences from *Mus musculus* and *Caenorhabditis elegans* formed distinct clusters as expected.

### RNA modifications in rG4 loci

Emerging evidence has shown that rG4s are tightly coupled with RNA modifications. Our results further support the idea that RNA bases carrying chemical modifications can alter the base-pairing capability of nucleotides, thereby impairing RNA structure equilibrium, as many PQSs can also fold into SL structures. Therefore, we collected RNA modification sites in transcripts, including mRNAs, lncRNAs, and miRNAs, from resources cataloged in GEO, RMBase, RMVar, MiREDiBase, and m^6^A-Atlas databases, as well as from published datasets. These modification sites were found within 15% and 13% of total PQSs in the human and mouse transcriptomes, respectively. The high-confidence m^6^A sites within PQSs, supported by experimental evidence at single-base resolution, represent 41% and 32% of the total PQSs that contain m^6^A sites in the human and mouse transcriptomes, corresponding to 14,137 and 7800 sites, respectively. Among human PQSs with modifications, only 1.6% were supported by rG4-seq (Figure 4F).

## Experimental validation

**Figure 5:**
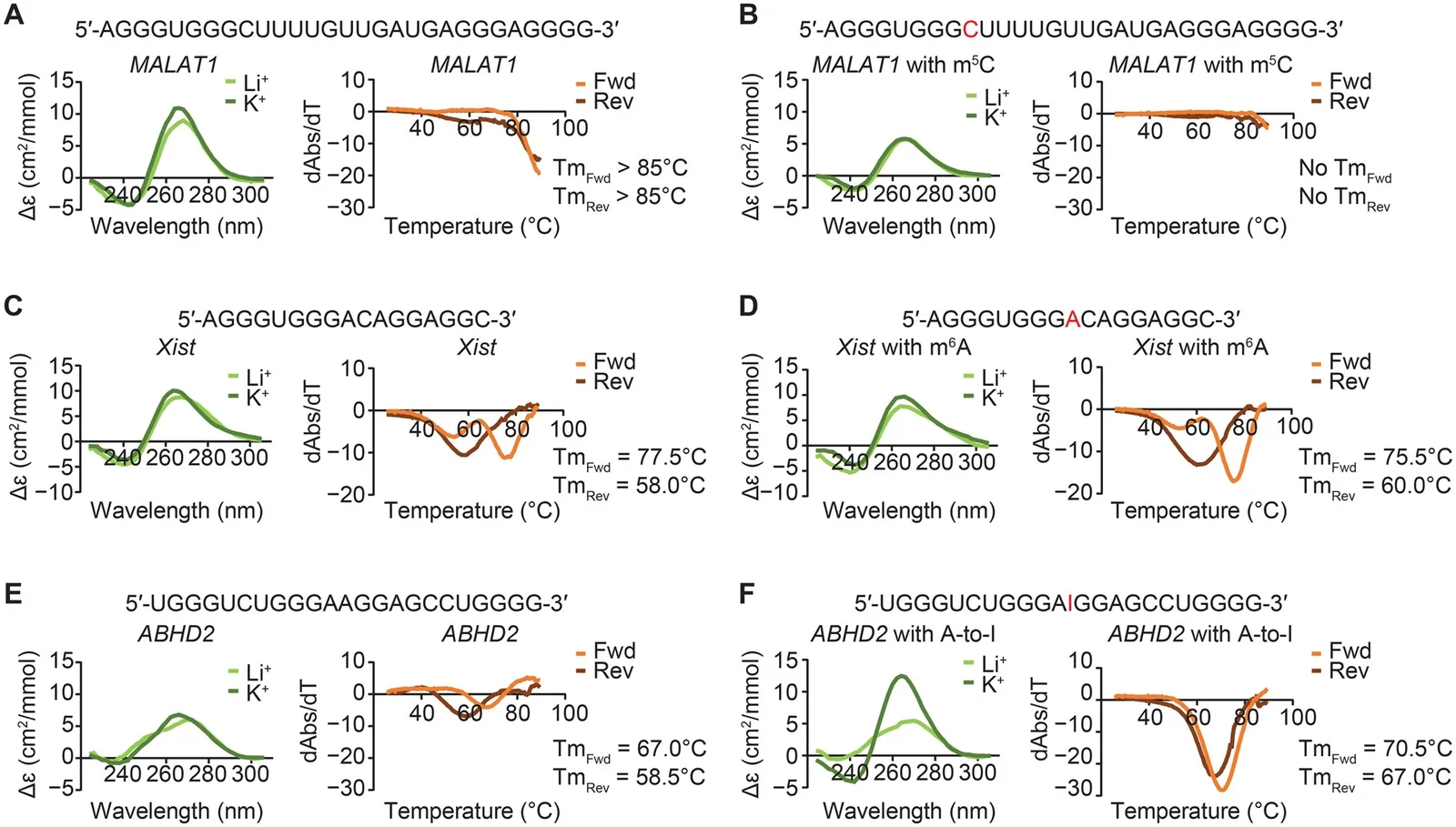
Biophysical characterization of PQSs in *MALAT1*, *Xist*,* ABHD2*, and their derivatives with RNA modifications **A**. Left: CD spectra of *MALAT1* under K^+^ and Li^+^ conditions. The CD pattern was stronger in the presence of K^+^ than in the presence of Li^+^, suggesting rG4 formation. The negative peak at 240 nm and the positive peak at 262 nm under K^+^ conditions suggest a parallel topology of rG4. Right: UV melting results of *MALAT1*. The hypochromic shift at 295 nm is a strong sign of rG4 formation. The forward experiments were conducted from 20°C to 95°C, while the reverse experiments were conducted from 95°C to 20°C. The available Tm values for each ligand are indicated. The corresponding oligo sequence is shown at the top. **B**. Left: CD spectra of *MALAT1* with m^5^C under K^+^ and Li^+^ conditions. Right: UV melting results of *MALAT1* with m^5^C. The corresponding oligo sequence is shown at the top, with the modification site in red. **C**. Left: CD spectra of *Xist* under K^+^ and Li^+^ conditions. Right: UV melting results of *Xist.* **D**. Left: CD spectra of *Xist* with m^6^A under K^+^ and Li^+^ conditions. Right: UV melting results of *Xist* with m^6^A. **E**. Left: CD spectra of *ABHD2* under K^+^ and Li^+^ conditions. Right: UV melting results of *ABHD2*. **F**. Left: CD spectra of *ABHD2* with A-to-I editing under K^+^ and Li^+^ conditions. Right: UV melting results of *ABHD2* with A-to-I editing. Tm, melting temperature; Fwd, forward; Rev, reverse.

To comprehensively demonstrate the impact of RNA modifications on secondary structure dynamics, we selected four PQSs from human and mouse transcripts carrying three types of modifications that are relatively abundant *in vivo*. Sequences from these loci include m^5^C in human *MALAT1* (supported by rG4-seq [56]), m^6^A in mouse *Xist* [identified by K^+^-dependent reverse transcription (RT)-stop profiling [10]], an A-to-I editing site in human *ABHD2* (lacking rG4-seq support), and m^5^C in mouse *Kmt2d* (containing multiple m^5^C sites supported by ultra-low-input rG4-seq [23]). All rG4-forming sequences were documented in MoRNiNG. For the *MALAT1* oligo, we found that m^5^C can potentially promote the formation of rG4 multimers or intermolecular rG4s (**Figure 5**A and B). However, the Tm could not be reliably determined for *MALAT1* oligos, probably due to the low rG4 concentration. Compared to the *Xist* oligo without m^6^A, the m^6^A-containing oligo exhibited a significantly decreased Tm in the forward UV melting scan (Figure 5C and D). A-to-I editing significantly enhanced rG4 formation with considerable thermostability (Figure 5E and F). We further explored the effect of one m^5^C *versus* two m^5^Cs in mouse *Kmt2d* and found that all tested oligos could form rG4s. However, compared to oligos without m^5^C, those containing m^5^C appeared to enhance rG4 multimerization or promote intermolecular rG4 formation, likely due to changes in intermolecular interactions induced by m^5^C modifications (Figure S13). These findings demonstrate that modifications can influence RNA conformation, further confirming the utility of this database.

## Discussion

The increasing volume of RNA modifications characterized at base resolution provides an opportunity to better understand the regulatory roles of RNAs, since chemical modifications to nucleotides alter base-pairing properties or the recognition of RBPs. However, the relationships between RNA structures and RNA modifications remain largely undefined. Beyond the known SL structure recognized by LIN28A, our experiments demonstrate that the non-canonical secondary structure, rG4, can also interact with this protein. rG4 can facilitate binding to LIN28A, regardless of the presence of potassium. For the existing structure profiling data of LIN28A, poor alignment between the reactivity score and SL can be explained by rG4 formation that competes with SL. Indeed, the UV melting experiment shows that m^6^A can reduce rG4 thermostability without drastically impairing the overall binding between RNA and LIN28A. This mechanism coincidentally explains LIN28A binding as an m^6^A anti-reader, with RNA modification acting as a switch [17]. Since the Mut1_GG2UU_ RNA exhibits a lower binding affinity for the protein than the original ligand, we could not rule out the possibility that LIN28A prefers binding to a certain form of rG4. Alternatively, a mixture of rG4s could somehow enhance the binding.

The compartmentalized distribution of K^+^ in cells [57] suggests that RNA conformation can dynamically change alongside ions within compartments in response to external cellular stress or stimuli, leading to a shift in the equilibrium of RNA conformation. In addition to the evidence that rG4s and RNA modifications are directly coupled [18], RNA modifications are commonly observed in neurons, where ion flux dictates cell excitability. Moreover, in several neurodegenerative diseases, including amyotrophic lateral sclerosis, frontotemporal dementia, and Alzheimer’s disease, RNA modifications are directly associated with disease-associated RBPs and their molecular crowding [15,16]. The dysregulated interaction between RNA and RBP suggests modulation of RNA structure by RNA modifications. RNA modifications alter the structural stability of RNAs, representing a potential regulatory layer that facilitates RNA structural dynamics, as supported by evidence from several tRNA modifications [13,58,59]. Our findings support a model in which RNA modifications, together with ions, impact the equilibrium of RNA secondary conformations to alter binding affinity for RBPs, thereby influencing subsequent regulatory processes. It is also possible that RNA modifications influence intermolecular interactions, as observed in cases with m^5^C modifications. Although elucidating the detailed mechanism underlying RNA stability shifts remains challenging, modification-induced changes in RNA structure could guide future drug discovery and potentially be combined with single-nucleotide polymorphisms to empower personalized medicine strategies.

The large proportion of RNA sequences with dual RNA structure-forming capacity in the genome suggests that the regulation of RNA structures requires greater focus during profiling experiments. This also points out another aspect of RNA folding: current RNA folding algorithms require additional parameters to expand their capability to predict and distinguish structural forms derived from the same RNA segment. Our study is limited to *in vitro* assays, providing proof of concept that RNA modification acts as a structure switch by shifting the equilibrium of distinct secondary structures [12]. A similar rule can potentially be extended to the relationship between unstructured RNA segments and structures, as indicated by A-to-I editing. Therefore, large-scale investigations, *in vivo* profiling, and better RNA folding algorithms would be of great importance for gaining a systematic view. However, carefully designed experiments and corresponding simulations demand cross-disciplinary collaborations, which are beyond our current research capacity. Leveraging the accumulated experimental evidence and large-scale data, we established the MoRNiNG database to help the RNA research community explore the roles of RNA modifications at single-base resolution within their associated rG4s via web browsers. This development opens the door to targeting rG4 regulation through RNA modifications. However, due to the scope and throughput of existing experimental assays, many candidate rG4 loci remain to be validated, and low-confidence loci should be used with caution. Improved rG4-seq protocols and tailored rG4 capturing assays that boost resolution and throughput would be valuable resources [23]. Advanced models or algorithms for the accurate identification of PQSs are also on the horizon [54,55,60]. Nevertheless, our study lays the groundwork for targeting rG4 regulation through RNA modifications.

In follow-up work, we will focus on several areas: (1) expanding the database capacity by including more RNA modifications from newly sequenced species and data types from other experiments to annotate PQSs, such as icSHAPE [61] and HTR-SELEX [6]; (2) incorporating or developing state-of-the-art algorithms to improve the precision of rG4 prediction, and introducing new tools to integrate RNA structure data to strengthen the confidence level of experimental evidence; (3) automating the data processing pipeline to implement a robust data curation procedure that quickly incorporates the latest experimental evidence and allows users to submit published datasets. Overall, the MoRNiNG database will continue to provide insights into the interplay between RNA epigenetics and structural dynamics.

## Supplementary Material

qzaf106_Supplementary_Data

## Data Availability

MoRNiNG can be accessed at https://www.cityu.edu.hk/bms/morning. MoRNiNG has been submitted to Database Commons [62] at the National Genomics Data Center (NGDC), China National Center for Bioinformation (CNCB), which is publicly accessible at https://ngdc.cncb.ac.cn/databasecommons/database/id/10241.

## CRediT author statement

**Yicen Zhou:** Data curation, Methodology, Investigation, Visualization, Software, Writing – original draft. **Shanxin Lyu:** Methodology, Investigation, Visualization, Writing – original draft. **Shiau Wei Liew:** Methodology, Investigation, Writing – review & editing. **Xi Mou:** Methodology, Investigation, Writing – review & editing. **Ian Hoffecker:** Investigation, Writing – review & editing. **Jian Yan:** Investigation, Writing – review & editing. **Yu Li**: Investigation, Writing – review & editing. **Chun Kit Kwok:** Conceptualization, Supervision, Investigation, Writing – review & editing. **Jilin Zhang:** Conceptualization, Investigation, Supervision, Investigation, Writing – original draft, review & editing. All authors have read and approved the final manuscript.
