# Supplementary material for: MoRNiNG: A Database of RNA Modification Sites Associated with RNA Secondary Structure Dynamics
Source: Genomics Proteomics Bioinformatics. 2025 Nov 14;24(2):qzaf106. doi: 10.1093/gpbjnl/qzaf106 (PMC13506053; doi:10.1093/gpbjnl/qzaf106)
Supplement: Supplementary file 1 [file qzaf106_supplementary_data.zip › Figure S10.pdf]

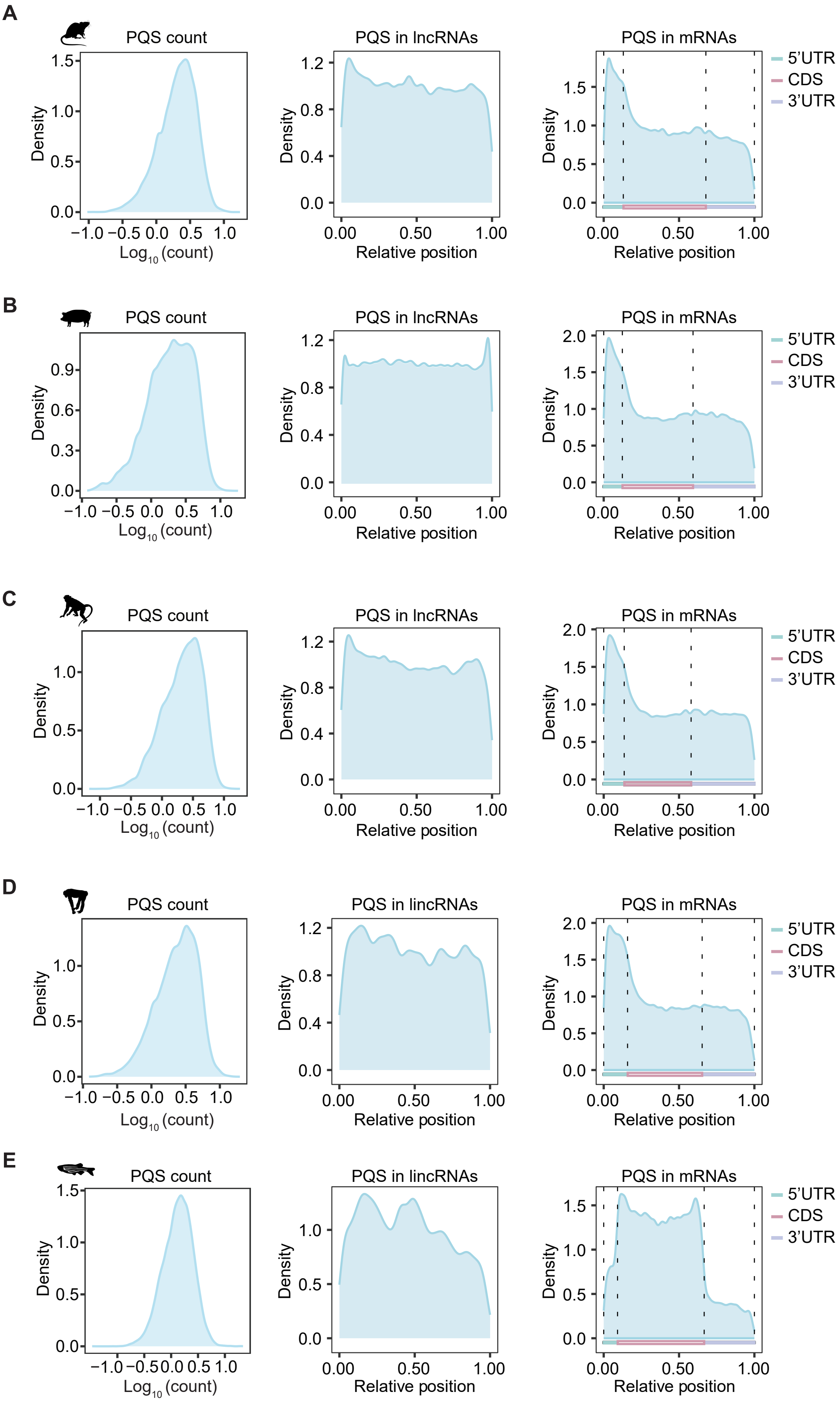

A
PQS count
Density
Log10(count)
PQS in lncRNAs
Density
Relative position
PQS in mRNAs
Density
Relative position
5'UTR
CDS
3'UTR
B
PQS count
PQS in lncRNAs
PQS in mRNAs
C
PQS count
PQS in lncRNAs
PQS in mRNAs
D
PQS count
PQS in lincRNAs
PQS in mRNAs
E
PQS count
PQS in lincRNAs
PQS in mRNAs
