# Supplementary material for: MoRNiNG: A Database of RNA Modification Sites Associated with RNA Secondary Structure Dynamics
Source: Genomics Proteomics Bioinformatics. 2025 Nov 14;24(2):qzaf106. doi: 10.1093/gpbjnl/qzaf106 (PMC13506053; doi:10.1093/gpbjnl/qzaf106)
Supplement: Supplementary file 1 [file qzaf106_supplementary_data.zip › Figure S11.pdf]

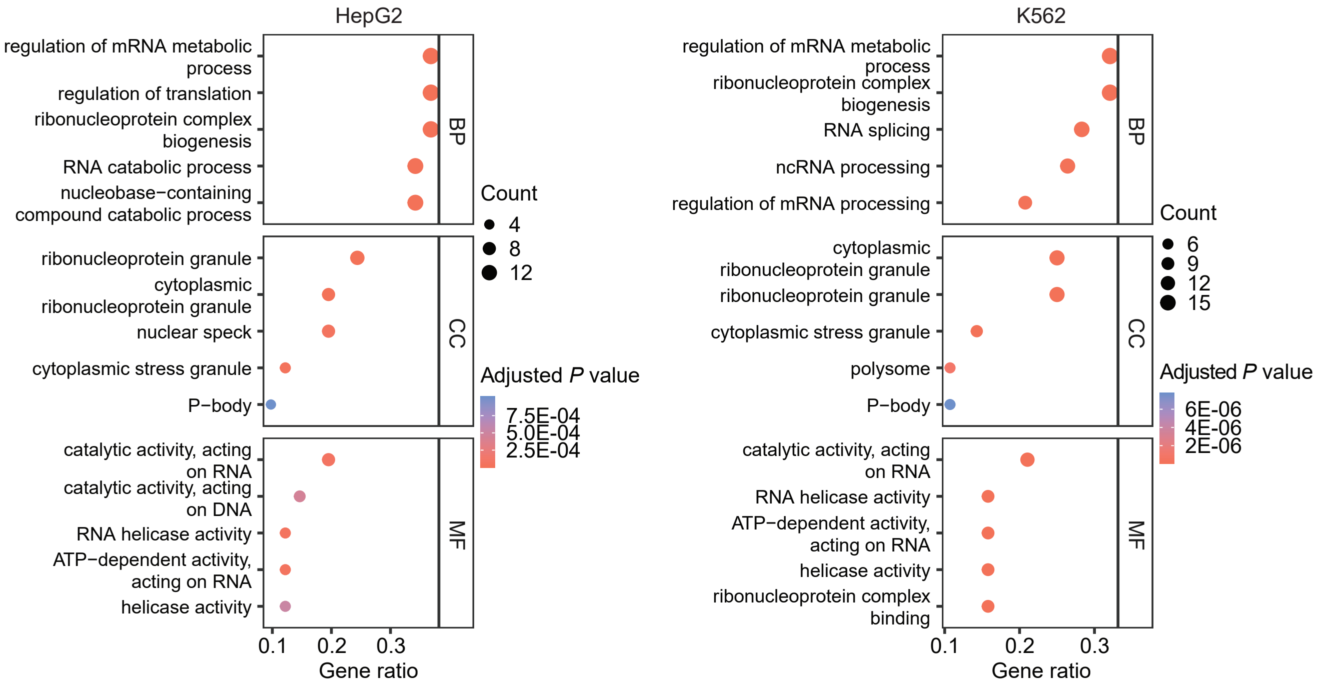
HepG2
regulation of mRNA metabolic process
regulation of translation
ribonucleoprotein complex biogenesis
RNA catabolic process
nucleobase−containing compound catabolic process
ribonucleoprotein granule
cytoplasmic ribonucleoprotein granule
nuclear speck
cytoplasmic stress granule
P−body
catalytic activity, acting on RNA
catalytic activity, acting on DNA
RNA helicase activity
ATP−dependent activity, acting on RNA
helicase activity
BP
CC
MF
0.1
0.2
0.3
Gene ratio
Count
4
8
12
Adjusted P value
7.5E-04
5.0E-04
2.5E-04
K562
regulation of mRNA metabolic process
ribonucleoprotein complex biogenesis
RNA splicing
ncRNA processing
regulation of mRNA processing
cytoplasmic ribonucleoprotein granule
ribonucleoprotein granule
cytoplasmic stress granule
polysome
P−body
catalytic activity, acting on RNA
RNA helicase activity
ATP−dependent activity, acting on RNA
helicase activity
ribonucleoprotein complex binding
BP
CC
MF
0.1
0.2
0.3
Gene ratio
Count
6
9
12
15
Adjusted P value
6E-06
4E-06
2E-06
