# Supplementary material for: MoRNiNG: A Database of RNA Modification Sites Associated with RNA Secondary Structure Dynamics
Source: Genomics Proteomics Bioinformatics. 2025 Nov 14;24(2):qzaf106. doi: 10.1093/gpbjnl/qzaf106 (PMC13506053; doi:10.1093/gpbjnl/qzaf106)
Supplement: Supplementary file 1 [file qzaf106_supplementary_data.zip › Figure S12.pdf]

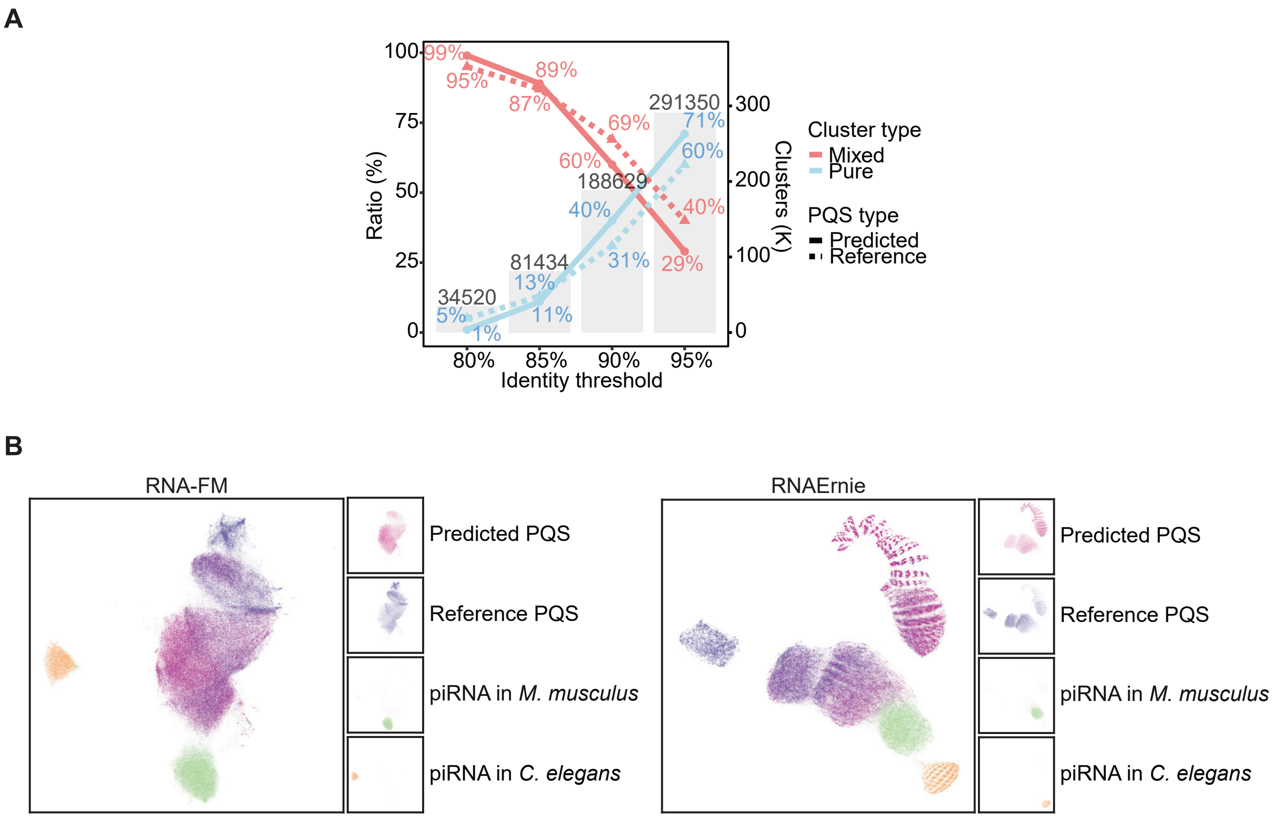

A
Ratio (%)
Clusters (K)
Identity threshold
80%
85%
90%
95%
34520
81434
188629
291350
99%
95%
89%
87%
69%
60%
40%
29%
71%
60%
40%
31%
13%
11%
5%
1%
Cluster type
Mixed
Pure
PQS type
Predicted
Reference
B
RNA-FM
RNAErnie
Predicted PQS
Reference PQS
piRNA in M. musculus
piRNA in C. elegans
