# Supplementary material for: MoRNiNG: A Database of RNA Modification Sites Associated with RNA Secondary Structure Dynamics
Source: Genomics Proteomics Bioinformatics. 2025 Nov 14;24(2):qzaf106. doi: 10.1093/gpbjnl/qzaf106 (PMC13506053; doi:10.1093/gpbjnl/qzaf106)
Supplement: Supplementary file 1 [file qzaf106_supplementary_data.zip › Figure S13.pdf]

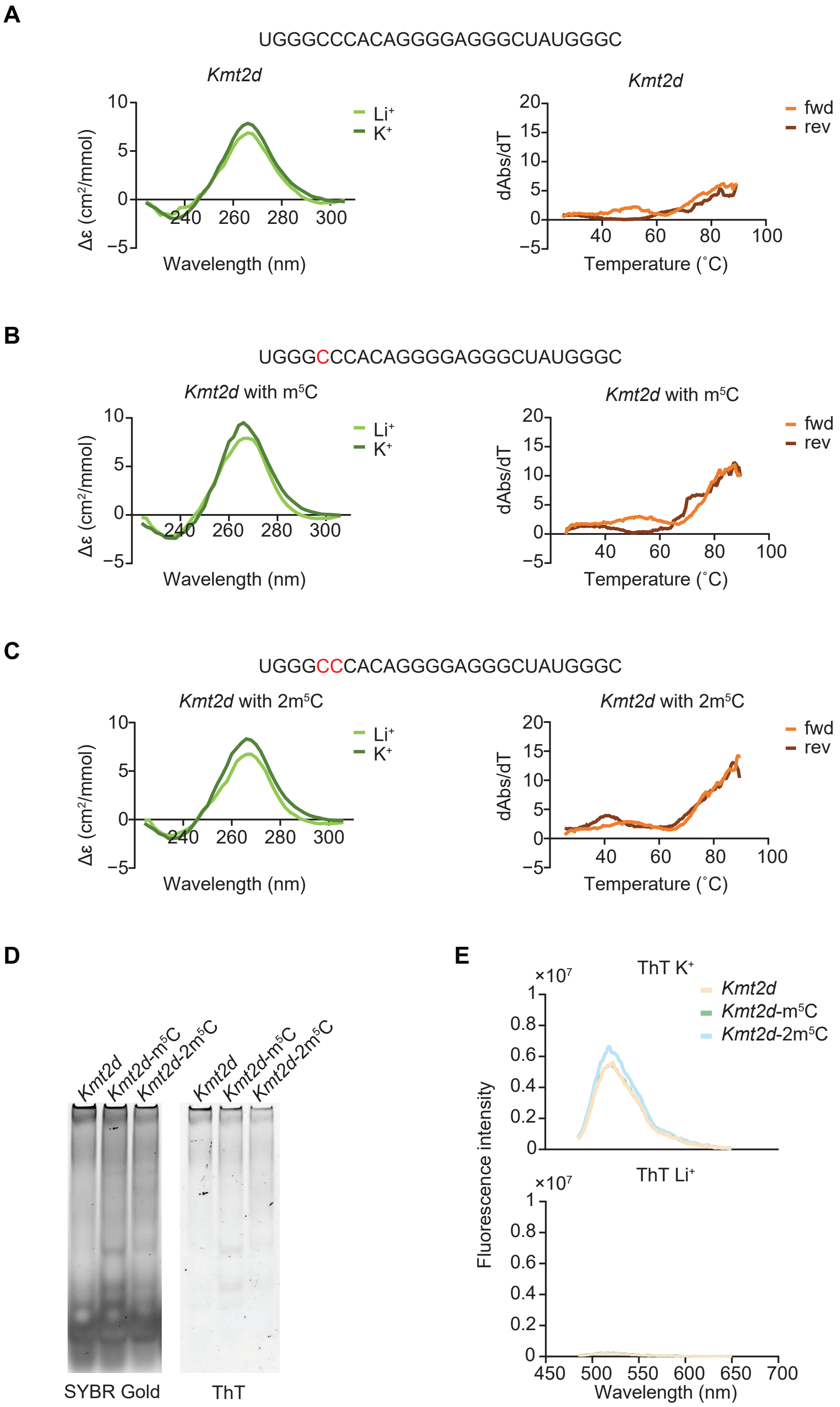
A
UGGGCCCACAGGGGAGGGCUAUGGGC
Kmt2d
Δε (cm²/mmol)
Wavelength (nm)
Li⁺
K⁺
Kmt2d
dAbs/dT
Temperature (˚C)
fwd
rev
B
UGGGCCCACAGGGGAGGGCUAUGGGC
Kmt2d with m⁵C
Δε (cm²/mmol)
Wavelength (nm)
Li⁺
K⁺
Kmt2d with m⁵C
dAbs/dT
Temperature (˚C)
fwd
rev
C
UGGGCCCACAGGGGAGGGCUAUGGGC
Kmt2d with 2m⁵C
Δε (cm²/mmol)
Wavelength (nm)
Li⁺
K⁺
Kmt2d with 2m⁵C
dAbs/dT
Temperature (˚C)
fwd
rev
D
Kmt2d
Kmt2d-m⁵C
Kmt2d-2m⁵C
Kmt2d
Kmt2d-m⁵C
Kmt2d-2m⁵C
SYBR Gold
ThT
E
ThT K⁺
×10⁷
Kmt2d
Kmt2d-m⁵C
Kmt2d-2m⁵C
Fluorescence intensity
ThT Li⁺
×10⁷
Wavelength (nm)
