# Supplementary material for: MoRNiNG: A Database of RNA Modification Sites Associated with RNA Secondary Structure Dynamics
Source: Genomics Proteomics Bioinformatics. 2025 Nov 14;24(2):qzaf106. doi: 10.1093/gpbjnl/qzaf106 (PMC13506053; doi:10.1093/gpbjnl/qzaf106)

Basic information of RNA modification site:

**Mod ID**	hsa_m6A_7405
**Mod type**	m6A
**Chromosome**	chr17
**Position**	48621735
**Strand**	-
**Transcript** ⓘ	ENST00000311177
**Symbol** ⓘ	HOXB9
**Site in transcript** ⓘ	A2007
**RNA type**	mRNA
**PQS with modfication** ⓘ	5'-GGGACAAGUGGUCUGGGACAGGG-3'
**PQS range** ⓘ	chr17:48621730-48621752
**PQS length**	23
**Site in PQS** ⓘ	A18
**Site type** ⓘ	loop
**Raw PQS and Optimal secondary structure** ⓘ	GGGACAAGUGGUCUGGGACAGGG .((((.....))))......... (-2.70)

Additional information:

**Modification detection method** ⓘ	GLORI:(High);m6A-seq:(Medium),MAZTER-seq:(High);Prediction by CNN model:(Low);miCLIP2&m6Aboost:(High);CITS_miCLIP:(High);miCLIP:(High)
**rG4 detection method** ⓘ	pqsfinder&qgrs;G4RNA screener:(Medium)
**Biological source**	HeLa,ESC-293T;293T;insulin-treated 293E;HEK293
**Data source**	RMBase database;34157120;26121403;33756105;m6A-Atlas database;RMVar database;36302990
**Publication** ⓘ	36302990;26121403;33756105;34157120;31257032;29507755

JBrowse

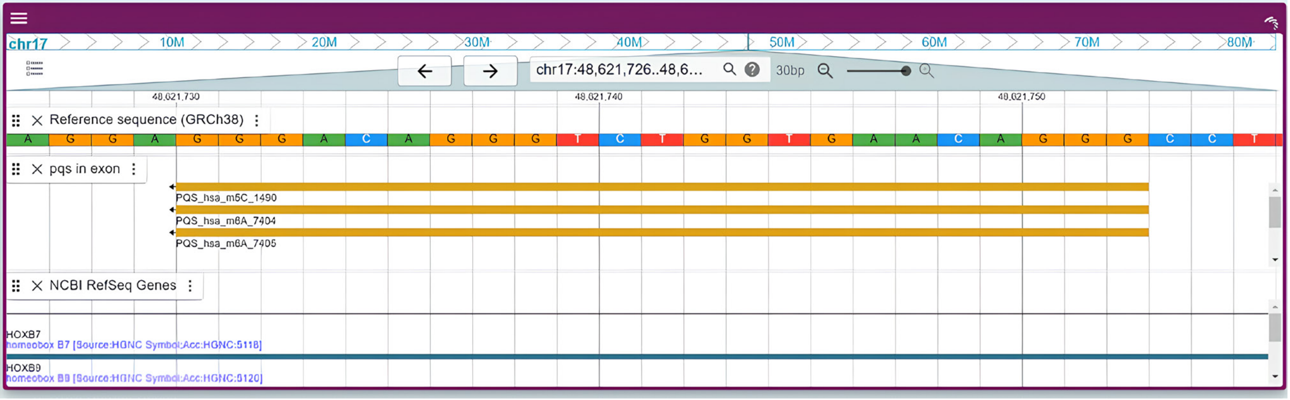

Supplement: Supplementary file 1 [file qzaf106_supplementary_data.zip › Figure S2.pdf]
