# Supplementary material for: MoRNiNG: A Database of RNA Modification Sites Associated with RNA Secondary Structure Dynamics
Source: Genomics Proteomics Bioinformatics. 2025 Nov 14;24(2):qzaf106. doi: 10.1093/gpbjnl/qzaf106 (PMC13506053; doi:10.1093/gpbjnl/qzaf106)
Supplement: Supplementary file 1 [file qzaf106_supplementary_data.zip › Figure S3.pdf]

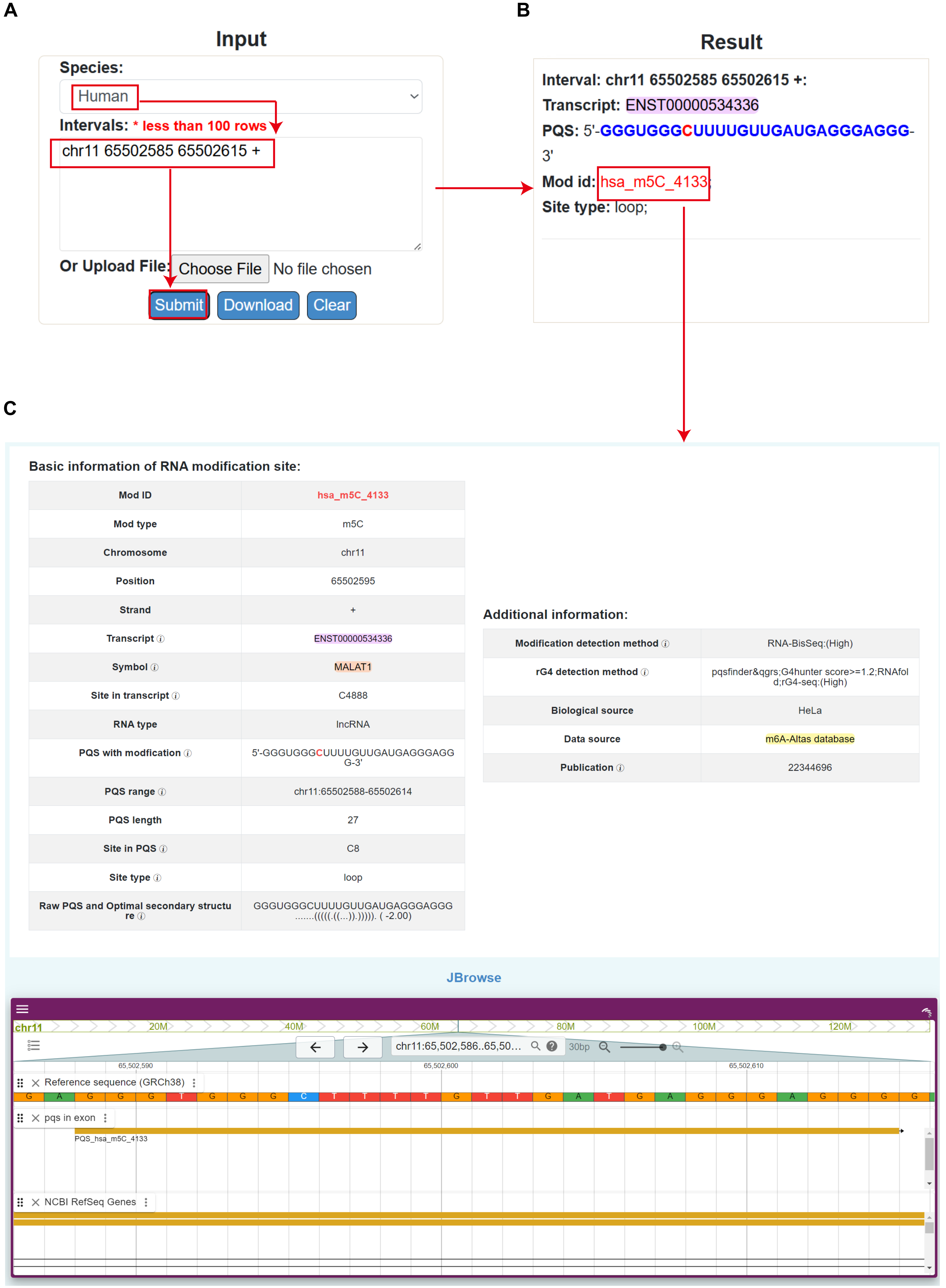
A
Input
Species:
Human
Intervals: * less than 100 rows
chr11 65502585 65502615 +
Or Upload File: Choose File No file chosen
Submit
Download
Clear
B
Result
Interval: chr11 65502585 65502615 +:
Transcript: ENST00000534336
PQS: 5'-GGGUGGGCUUUUGUUGAUGAGGGAGGG-3'
Mod id: hsa_m5C_4133;
Site type: loop;
C
Basic information of RNA modification site:
Mod ID hsa_m5C_4133
Mod type m5C
Chromosome chr11
Position 65502595
Strand +
Transcript ENST00000534336
Symbol MALAT1
Site in transcript C4888
RNA type lncRNA
PQS with modfication 5'-GGGUGGGCUUUUGUUGAUGAGGGAGGG-3'
PQS range chr11:65502588-65502614
PQS length 27
Site in PQS C8
Site type loop
Raw PQS and Optimal secondary structure GGGUGGGCUUUUGUUGAUGAGGGAGGG .......(((((.((...)).))))). (-2.00)
Additional information:
Modification detection method RNA-BisSeq:(High)
rG4 detection method pqsfinder&qgrs;G4hunter score>=1.2;RNAfold;rG4-seq:(High)
Biological source HeLa
Data source m6A-Altas database
Publication 22344696
JBrowse
chr11
20M
40M
60M
80M
100M
120M
chr11:65,502,586..65,50…
30bp
65,502,590
65,502,600
65,502,610
Reference sequence (GRCh38)
G A G G G T G G G C T T T T G T T G A T G A G G G A G G G
pqs in exon
PQS_hsa_m5C_4133
NCBI RefSeq Genes
