# Supplementary material for: MoRNiNG: A Database of RNA Modification Sites Associated with RNA Secondary Structure Dynamics
Source: Genomics Proteomics Bioinformatics. 2025 Nov 14;24(2):qzaf106. doi: 10.1093/gpbjnl/qzaf106 (PMC13506053; doi:10.1093/gpbjnl/qzaf106)

A

Algorithm	PQS	Score	G-tetrad labelled
G4RNA screener	GGGACAAGUGGUCUGGGACAGGGAGGAGCAAC	8,1,0.92	No
G4Hunter	GGGACAAGUGGUCUGGGACAGGGAGG	1.27	No
pqsfinder	GGGACAAGUGGUCUGGGACAGGG	43	Yes
QGRS	GGGACAAGUGGUCUGGGACAGGG	19	Yes
HOXB9 oligo	GGGACAAGUGGUCUGGGACAGGGAGGAGCAAC		

G-tetrad labelled: Yes / No

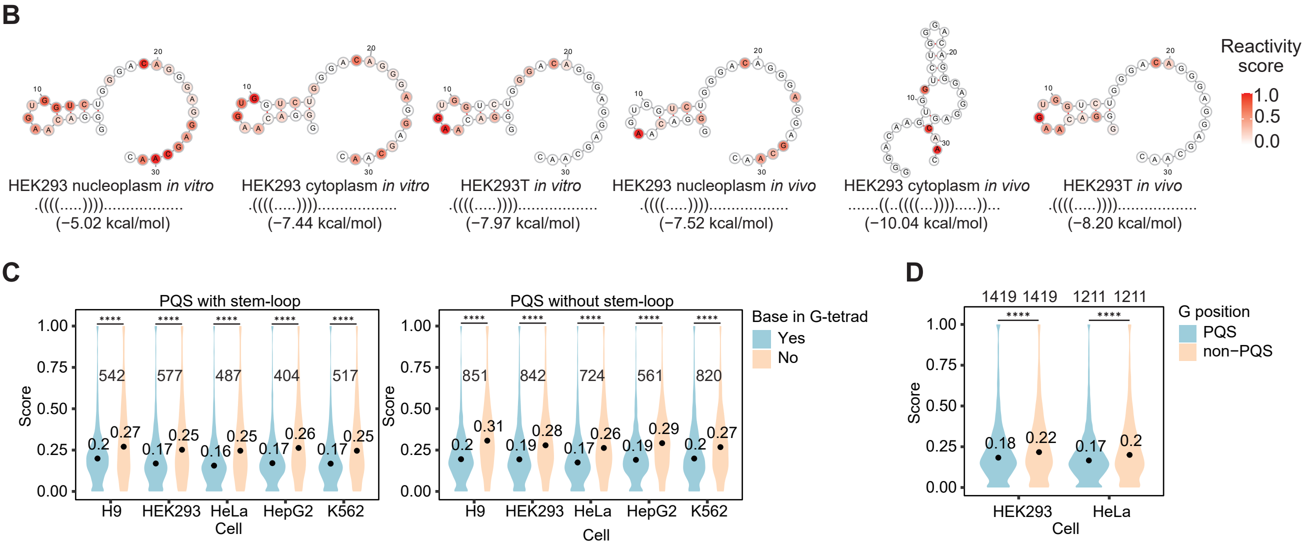

Supplement: Supplementary file 1 [file qzaf106_supplementary_data.zip › Figure S4.pdf]
