# Supplementary material for: MoRNiNG: A Database of RNA Modification Sites Associated with RNA Secondary Structure Dynamics
Source: Genomics Proteomics Bioinformatics. 2025 Nov 14;24(2):qzaf106. doi: 10.1093/gpbjnl/qzaf106 (PMC13506053; doi:10.1093/gpbjnl/qzaf106)
Supplement: Supplementary file 1 [file qzaf106_supplementary_data.zip › Figure S6.pdf]

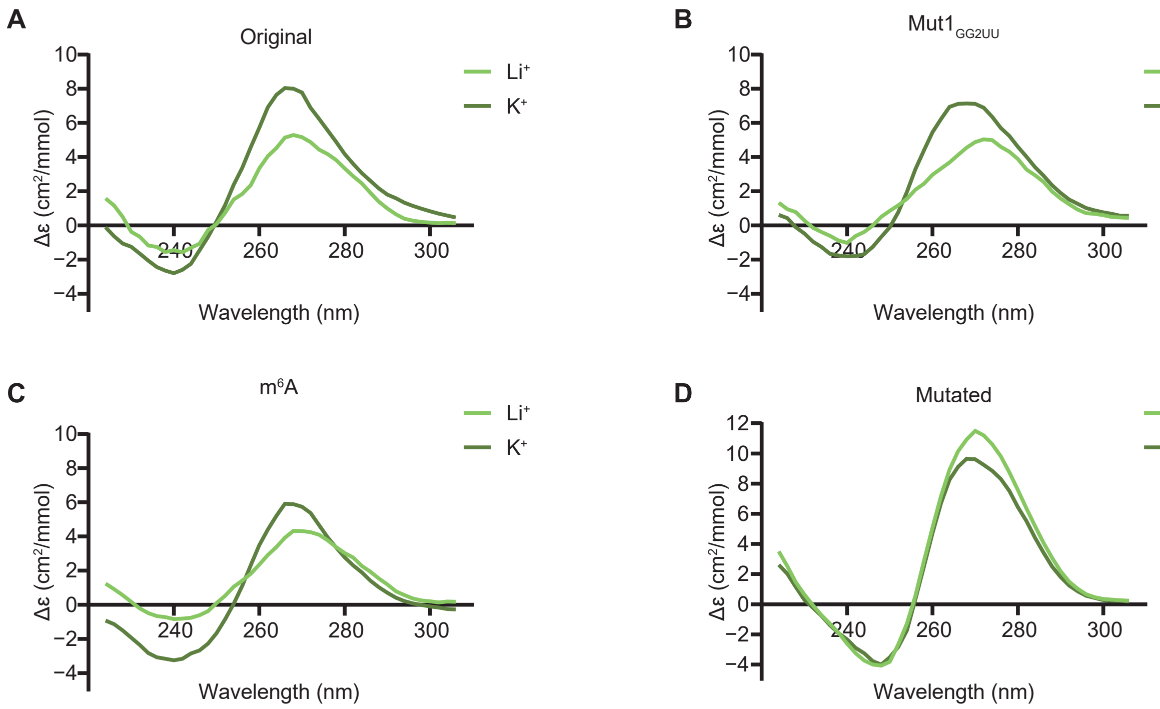
A
Original
B
Mut1$_{GG2UU}$
C
m^{6}A
D
Mutated
Li$^+$
K$^+$
Δε (cm^2/mmol)
Wavelength (nm)
