# Supplementary material for: MoRNiNG: A Database of RNA Modification Sites Associated with RNA Secondary Structure Dynamics
Source: Genomics Proteomics Bioinformatics. 2025 Nov 14;24(2):qzaf106. doi: 10.1093/gpbjnl/qzaf106 (PMC13506053; doi:10.1093/gpbjnl/qzaf106)
Supplement: Supplementary file 1 [file qzaf106_supplementary_data.zip › Figure S7.pdf]

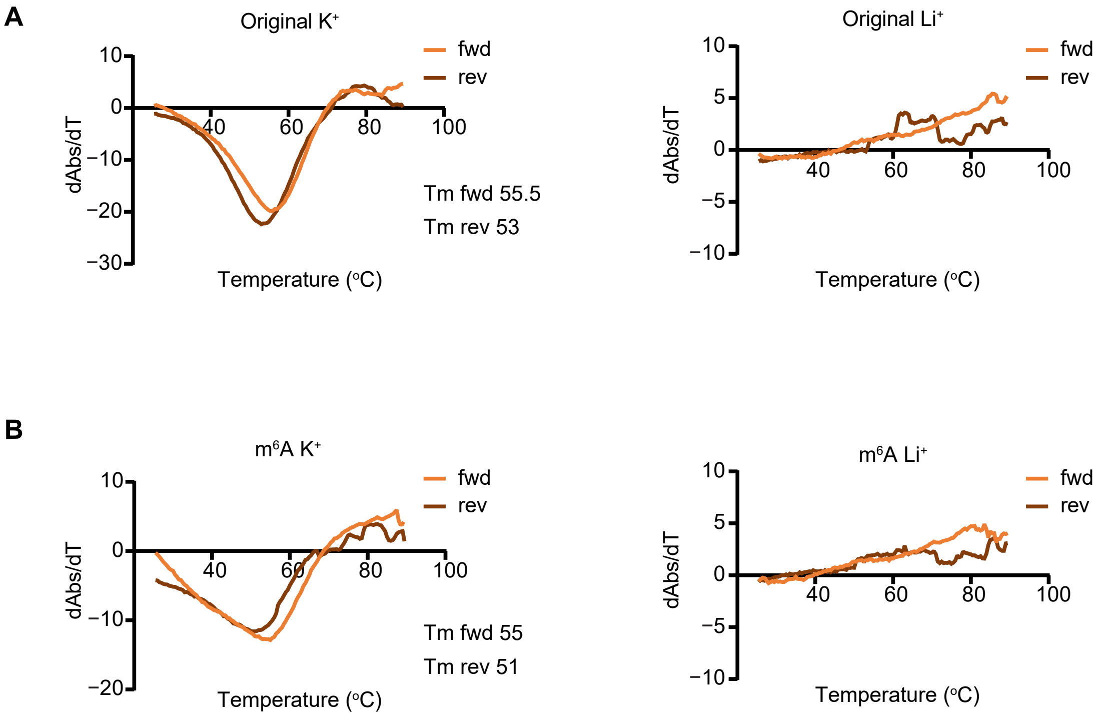

A
Original K+
fwd
rev
Tm fwd 55.5
Tm rev 53
dAbs/dT
10
0
−10
−20
−30
40
60
80
100
Temperature (°C)
Original Li+
fwd
rev
dAbs/dT
10
5
0
−5
−10
40
60
80
100
Temperature (°C)
B
m6A K+
fwd
rev
Tm fwd 55
Tm rev 51
dAbs/dT
10
0
−10
−20
40
60
80
100
Temperature (°C)
m6A Li+
fwd
rev
dAbs/dT
10
5
0
−5
−10
40
60
80
100
Temperature (°C)
