# Supplementary material for: MoRNiNG: A Database of RNA Modification Sites Associated with RNA Secondary Structure Dynamics
Source: Genomics Proteomics Bioinformatics. 2025 Nov 14;24(2):qzaf106. doi: 10.1093/gpbjnl/qzaf106 (PMC13506053; doi:10.1093/gpbjnl/qzaf106)
Supplement: Supplementary file 1 [file qzaf106_supplementary_data.zip › Figure S8.pdf]

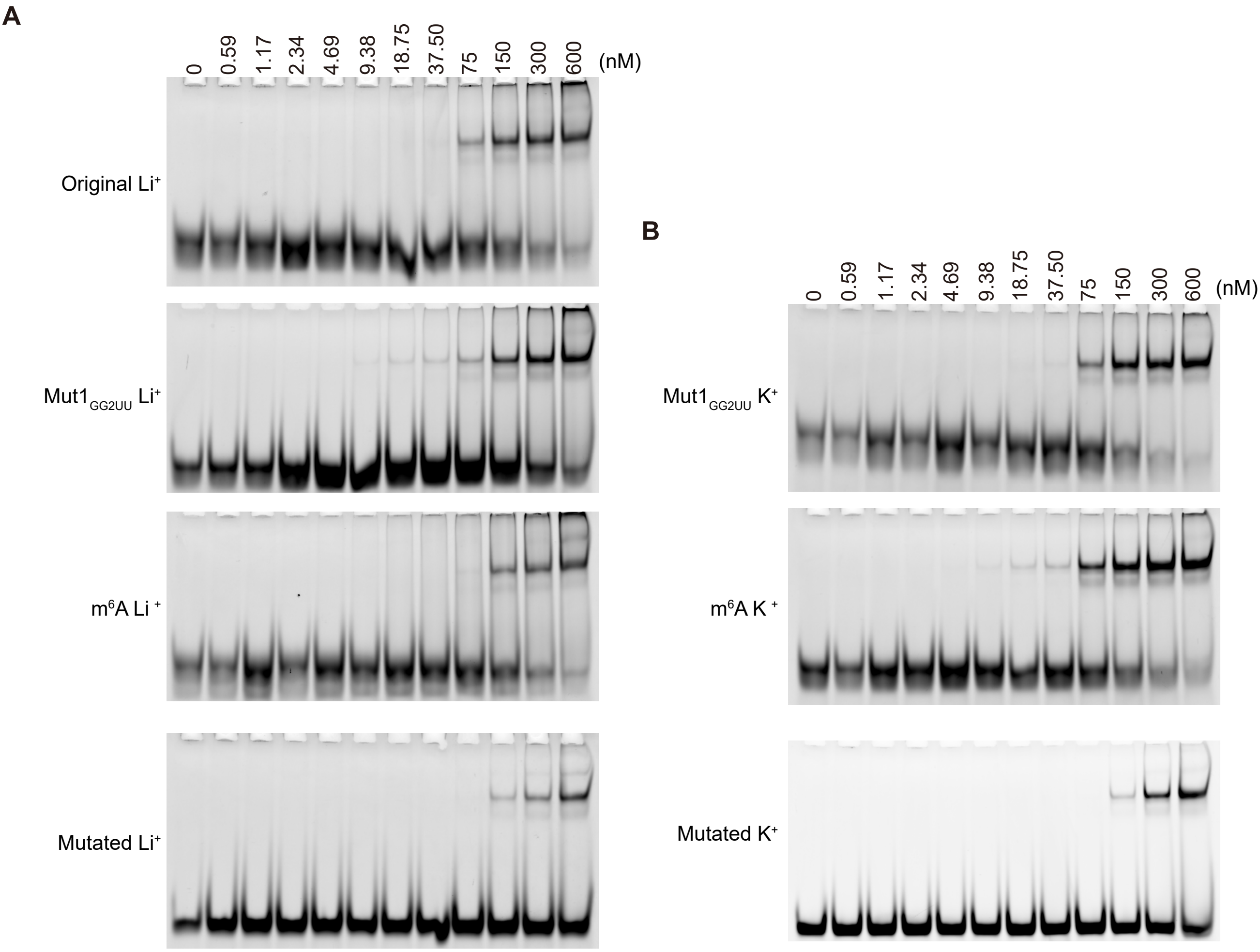
A
0
0.59
1.17
2.34
4.69
9.38
18.75
37.50
75
150
300
600
(nM)
Original Li⁺
Mut1 GG2UU Li⁺
m6A Li ⁺
Mutated Li⁺
B
0
0.59
1.17
2.34
4.69
9.38
18.75
37.50
75
150
300
600
(nM)
Mut1 GG2UU K⁺
m6A K ⁺
Mutated K⁺
