# Supplementary material for: MoRNiNG: A Database of RNA Modification Sites Associated with RNA Secondary Structure Dynamics
Source: Genomics Proteomics Bioinformatics. 2025 Nov 14;24(2):qzaf106. doi: 10.1093/gpbjnl/qzaf106 (PMC13506053; doi:10.1093/gpbjnl/qzaf106)
Supplement: Supplementary file 1 [file qzaf106_supplementary_data.zip › File S1.docx]

**Supplementary method**

**Oligonucleotides and G4-binding small molecules**

The original human *HOXB9* sequence and its derivatives were synthesized with and without the fluorescein amidite (FAM) tag on the 5′ end from Sangon (Shanghai, China) and Integrated DNA Technologies (IDT) (Coralville, IA), respectively. They were dissolved to a concentration of 100 μM as instructed by the manufacturers with ultra-pure nuclease-free distilled water (catalog No. 10977015, Thermo, Waltham, MA). All the dissolved oligos were stored at −20℃ before the experiment. Thioflavin T (ThT, catalog No. HY-D0218) and *N*-methyl mesoporphyrin IX (NMM, Catalog No. HY-133821) were ordered from MedChemExpress (Shanghai, China). SYBR Gold was ordered from Invitrogen (Catalog No. S11494, Waltham, MA).

**ThT and SYBR Gold native gel staining of rG4**

Reaction mixtures containing 10 mM Tris-HCl (pH 7.5), 150 mM KCl, and 5 μM oligonucleotide were prepared in a 20 μl reaction. The mixtures were heated at 95℃ for 5 min for denaturation and cooled to room temperature for 15 min. 3 µl of 40% sucrose (final ~5%) was added to the mixtures for gel loading. The mixtures were loaded into a 10% native polyacrylamide gel (Acr-Bis 19:1) with 5–10 µl of volume per well. The gel was run at 300 V at 4℃ for 20 min. For the ThT staining gel, the gel was stained with 0.32 µg/ml (1 μM final) ThT for 5 min by adding 5 µl of 10 mM ThT and 50 ml of milli-Q water. For SYBR Gold staining, the gel was stained with 1× SYBR Gold for 5 min by adding 5 µl of 10000 × SYBR Gold and 50 ml of milli-Q water. The gels were scanned with SYBR Gold mode using Biorad ChemDoc imaging system (Hercules, CA).

**Ligand-enhanced fluorescence assay**

Sample solutions containing 0.5 μM RNA were prepared in 150 mM LiCl/KCl and 0.5 or 1 μM ligand (NMM/ThT). Fluorescence spectroscopy was performed using a Molecular Devices SpectraMax ID5 microplate reader (San Jose, CA), and a 96-well plate was used with a sample volume of 150 μl. Before the measurement, the samples (ligand not added) were denatured at 95℃ for 3 min and allowed to cool at room temperature for 15 min. The samples were excited at 395 nm for NMM and 425 nm for ThT. The emission spectra were acquired from 550 to 750 nm and 465 (or 485) to 650 nm for NMM and ThT, respectively. Data were collected every 1 nm at 25℃.

**Circular dichroism spectroscopy**

Circular dichroism (CD) assay was performed with a Jasco CD J-150 spectrometer (Tokyo, Japan) and a 1 cm path length quartz cuvette. The reaction samples containing 5 μM oligos were prepared in 10 mM LiCac buffer pH 7.0, and 150 mM KCl/LiCl to form a total volume of 2 ml. The mixtures were then vortexed and heated at 95 ℃ for 5 min and cooled down to room temperature for 15 min for renaturation. The samples were scanned at a 2 nm interval from 220 to 310 nm, and the data were blanked and normalized to mean residue ellipticity before being smoothed over 8 nm.

**UV melting assay**

UV melting assay was performed using a Cary 100 UV-vis spectrophotometer (Santa Clara, CA) and a 1 cm path length quartz cuvette. The reaction samples containing 5 μM oligos were separately prepared in 10 mM LiCac buffer at pH 7.0 and 150 mM KCl to form a total volume of 2 ml. The mixtures were vortexed and heated at 95°C for 5 min and allowed to cool for 15 min at room temperature for renaturation. In cuvettes sealed with Teflon tape, the samples were monitored at 295 nm from 20°C to 95°C with data collected every 0.5°C. The data were blanked and smoothed over every 10°C.

**LIN28A protein purification**

The *LIN28A* gene and His-tag were inserted into the pET-28ɑ vector and then cloned into *Escherichia coli* BL21 (DE3). Bacteria were cultured in Luria Bertani (LB) broth medium with 100 μg/ml kanamycin overnight at 37°C on a shaker. LB included 0.5% (w/v) yeast extract, 1.0% (w/v) Tryptone, and 0.5% (w/v) NaCl in distilled water (pH 7.2). The culture was inoculated (1:100) into a fresh LB broth until OD600 = 0.5–0.6 was reached. To induce protein synthesis, 0.25 mM IPTG was added to the culture, which was then shaken overnight at 37°C. Cells were collected by centrifugation at 4000 rpm for 30 min. Then, cells were lysed and disrupted by 3 cycles of freezing (−80°C), thawing (37°C), and sonication. Afterward, the mixture was centrifuged at 4000 × g for 20 min, and the clear supernatant was passed through a 0.45 μm filter. Subsequently, protein purification was performed using BioRad Ni-charged resin (Catalog No. 1560125, Hercules, CA) for His-tagged recombinant proteins (in the denaturing condition) according to the manufacturer’s recommendation. Protein purity was evaluated by 10% SDS-PAGE. Finally, the concentration of proteins was determined by Thermo Narop Spectrophotometers (Waltham, MA).

**Electrophoretic mobility shift assay**

RNAs were heated at 95℃ for 3 min for denaturation and cooled to room temperature for 15 min. Reaction mixtures containing 10 nM 5′ FAM-labeled RNA, 20 mM HEPES pH 7.5, 100 mM KCl (stable rG4) or 100 mM LiCl (unstable rG4), 0.12 mM EDTA, 25 mM Tris-HCl (pH 7.5), 10% glycerol, and increasing concentrations of LIN28A were prepared in 10 μl and incubated at 37℃ for 1 h. The RNA–protein mixture was resolved by 10%, 37.5:1 (acrylamide: bis-acrylamide) non-denaturing polyacrylamide gel in 0.5 × Tris/Borate/EDTA (TBE) at 4℃, under the setting of 100 V for 30 min or 150 V for 40 min.

**Hierarchical annotation of candidate PQSs and rG4s**

Putative G-quadruplex-forming sequences (PQSs) were primarily predicted within the longest transcript through pqsfinder (minimum score = 1) and QGRS Mapper (default parameters). The candidate PQSs were processed by a customized Python script to extract the consensus PQSs, which were further fed into G4Hunter, G4RNA screener, and RNAfold to increase the reliability. In addition to the core PQSs predicted by the two programs, regions were labeled as “G4Hunter score ≥ 1.2” for PQS or RNA G-quadruplex (rG4), where G4Hunter results met the parameter settings “-w 25 -s 1.2”. These regions were further annotated with the supporting source “G4RNA Screener”, but only if the consecutive G over consecutive C ratio (cGcC), G4H, and G4NN scores were greater than or equal to 4.5, 0.9, and 0.5, respectively. RNAfold was additionally employed to examine whether PQSs could form rG4. One PQS was considered experimentally supported if it was completely covered by rG4-seq peaks from HeLa cells or if the terminal G of the PQS was located either at the reverse transcription stop (RTS) site or 1nt upstream of rG4-seq 2.0 of HEK293T cells [1] or rG4-seq of HeLa cells [2]. PQSs on mouse transcripts were additionally examined by intersecting with rG4 peaks identified by ultra-low-input rG4-seq experiments [3].

**SL structure prediction and icSHAPE reactivity score distribution of the *HOXB9* ligand**

The icSHAPE reactivity scores of the *HOXB9* sequence were downloaded from the RASP database, using the experiments conducted in HEK293T and HEK293 cell lines [4]. The data were grouped into *in vivo* and *in vitro* categories according to the experimental methods. The icSHAPE scores were subjected to RNAfold to predict the secondary structure [5]. The heatmap was visualized using the ComplexHeatmap [6]. The score of guanines in G-tetrad and other bases from the above six samples was examined.

**icSHAPE reactivity score distribution of PQSs supported by rG4-seq and non-PQSs**

For human PQSs supported by rG4-seq, only those containing at least 1nt in any loop and no bulges/mismatches in G-tetrads were considered. The corresponding imputed icSHAPE scores of H9, HEK293, HeLa, K562, and HepG2 cell lines were extracted from the RASP database [4]. PQSs with null reactivity scores at any position were further excluded. Scores of bases within G-tetrads were compared with those of bases in loops. Additionally, an equal number of non-PQS RNA fragments with the same length as sampled PQSs were randomly selected from HEK293 and HeLa cells to compare the score of guanines. The score difference between groups was examined by the Mann-Whitney test. Statistical significance was defined as follows: *, *P* < 0.05; **, *P* < 0.01; ***, *P* < 0.001; ****, *P* < 0.0001.

**Gene ontology enrichment analysis**

To explore the functional roles of potential rG4-binding RBPs, transposable elements (TEs)-free eCLIP peaks overlapping with PQSs were defined as rG4-binding sites for each RBP in HepG2 and K562 cell lines. The number of rG4-RBP peaks was then divided by the total count of non-TE peaks to get the ratio of rG4-peaks for each RBP. The gene ontology (GO) enrichment analysis was performed on RBPs with an rG4-peak ratio higher than 15% using clusterProfiler [7]. In total, 41 and 57 rG4-binding RBPs were defined in HepG2 and K562 cells, respectively.

**PQSs in eCLIP peaks**

The PQSs within the longest mRNA and lncRNA transcripts of the human transcriptome were intersected with eCLIP data (GRCh38) of K562 and HepG2 cell lines from the ENCODE database [8]. First, the peaks of distinct replicates were merged for each RBP with BEDTools [9]. The merged peaks were then intersected with PQS coordinates to obtain the peaks covering at least 50% of nucleotides in the overlapping PQSs. After removing duplicated PQSs, the proportion of PQSs supported by eCLIP peaks in the overall PQSs was calculated.

**PQSs supported by rG4-seq**

PQSs supported by rG4-seq were identified using the following criteria: PQSs completely covered by rG4-seq peaks or with the last G falling into the rG4-seq and rG4-seq 2.0 RTS sites or 1nt upstream. In the human transcriptome, PQSs supported by rG4-seq were counted by merging evidence from all available experimental sources, considering the low count of cell-specific peaks. For the PQS presenting RNA modification, only consistent sources for both RNA modification and rG4-seq (HeLa) and rG4-seq 2.0 (HEK293T) were considered.

**Comparison of PQSs with public G4 databases**

CD-HIT-EST [10] was used to cluster the 178,974 unique human PQSs located on mRNAs and lncRNAs, with 343,185 unique PQSs documented in QUADRatlas [11] and G4Atlas [12] (10nt ≤ length ≤ 60nt). Four sequence identity thresholds were applied: 80%, 85%, 90%, and 95%, corresponding to word sizes 5, 6, 9, and 10, respectively.

To compare higher-dimensional features beyond sequence similarity, 116,846 non-redundant PQSs were prepared using CD-HIT-EST with a sequence identity threshold of 90% to remove duplicated sequences. Applying the same approach to PQSs downloaded from QUADRatlas and G4Atlas resulted in 129,269 unique sequences. In addition, 48,484 *Mus musculus* piRNA sequences and 16,203 *Caenorhabditis* *elegans* piRNA sequences (10nt ≤ length ≤ 60nt) were taken from RNAcentral [13] as controls. These four sequence families were sent to RNA-FM [14] and RNAErnie [15] models, where the mean embeddings of each sequence were extracted to represent high-dimensional features. Finally, uniform manifold approximation and projection (UMAP) was applied to reduce 640-dimensional or 768-dimensional vectors to two dimensions for visualization [16].

**References**

[1]	Zhao J, Chow EY, Yeung PY, Zhang QC, Chan TF, Kwok CK. Enhanced transcriptome-wide RNA G-quadruplex sequencing for low RNA input samples with rG4-seq 2.0. BMC Biol 2022;20:257.

[2]	Chow EY, Lyu K, Kwok CK, Chan TF. rG4-seeker enables high-confidence identification of novel and non-canonical rG4 motifs from rG4-seq experiments. RNA Biol 2020;17:903–17.

[3]	Sun Q-Y, Meng T-G, Yue W, Li C, Gao D, Zongchang DU, et al. Ultra-low-input rG4-seq reveals the RNA G-quadruplex regulome in gene expression and genome integrity. Research Square 2024.

[4]	Li P, Zhou X, Xu K, Zhang QC. RASP: an atlas of transcriptome-wide RNA secondary structure probing data. Nucleic Acids Res 2021;49:D183–91.

[5]	Lorenz R, Bernhart SH, Honer Zu Siederdissen C, Tafer H, Flamm C, Stadler PF, et al. ViennaRNA Package 2.0. Algorithms Mol Biol 2011;6:26.

[6]	Gu Z, Eils R, Schlesner M. Complex heatmaps reveal patterns and correlations in multidimensional genomic data. Bioinformatics 2016;32:2847–9.

[7]	Xu S, Hu E, Cai Y, Xie Z, Luo X, Zhan L, et al. Using clusterProfiler to characterize multiomics data. Nat Protoc 2024;19:3292–320.

[8]	The ENCODE Project Consortium. An integrated encyclopedia of DNA elements in the human genome. Nature 2012;489:57–74.

[9]	Quinlan AR, Hall IM. BEDTools: a flexible suite of utilities for comparing genomic features. Bioinformatics 2010;26:841–2.

[10]	Fu L, Niu B, Zhu Z, Wu S, Li W. CD-HIT: accelerated for clustering the next-generation sequencing data. Bioinformatics 2012;28:3150–2.

[11]	Bourdon S, Herviou P, Dumas L, Destefanis E, Zen A, Cammas A, et al. QUADRatlas: the RNA G-quadruplex and rG4-binding proteins database. Nucleic Acids Res 2023;51:D240–7.

[12]	Yu H, Qi Y, Yang B, Yang X, Ding Y. G4Atlas: a comprehensive transcriptome-wide G-quadruplex database. Nucleic Acids Res 2023;51:D126–34.

[13]	Consortium TR, Petrov AI, Kay SJE, Kalvari I, Howe KL, Gray KA, et al. RNAcentral: a comprehensive database of non-coding RNA sequences. Nucleic Acids Res 2017;45:D128–34.

[14]	Shen T, Hu Z, Sun S, Liu D, Wong F, Wang J, et al. Accurate RNA 3D structure prediction using a language model-based deep learning approach. Nat Methods 2024;21:2287–98.

[15]	Wang N, Bian J, Li Y, Li X, Mumtaz S, Kong L, et al. Multi-purpose RNA language modelling with motif-aware pretraining and type-guided fine-tuning. Nat Mach Intell 2024;6:548–57.

[16]	McInnes L, Healy J, Saul N, Großberger L. UMAP: uniform manifold approximation and projection. J Open Source Softw 2018;3.
